# Circadian-driven tissue specificity is constrained under caloric restricted feeding conditions

**DOI:** 10.1038/s42003-024-06421-0

**Published:** 2024-06-20

**Authors:** Renrui Chen, Ziang Zhang, Junjie Ma, Bing Liu, Zhengyun Huang, Ganlu Hu, Ju Huang, Ying Xu, Guang-Zhong Wang

**Affiliations:** 1 CAS Key Laboratory of Computational Biology, Shanghai Institute of Nutrition and Health, University of Chinese Academy of Sciences, Chinese Academy of Sciences, Shanghai, 200031 China; 2 Collaborative Innovation Center for Brain Science, Department of Anatomy and Physiology, Shanghai Jiao Tong University School of Medicine, Shanghai, 200025 China; 3 Jiangsu Key Laboratory of Neuropsychiatric Diseases and Cambridge-Su Genomic Resource Center, Medical School of Soochow University, Suzhou, Jiangsu 215123 China; 4 Shanghai Institute for Advanced Immunochemical Studies, ShanghaiTech University, Shanghai, China

**Keywords:** Data integration, Transcriptomics

## Abstract

Tissue specificity is a fundamental property of an organ that affects numerous biological processes, including aging and longevity, and is regulated by the circadian clock. However, the distinction between circadian-affected tissue specificity and other tissue specificities remains poorly understood. Here, using multi-omics data on circadian rhythms in mice, we discovered that approximately 35% of tissue-specific genes are directly affected by circadian regulation. These circadian-affected tissue-specific genes have higher expression levels and are associated with metabolism in hepatocytes. They also exhibit specific features in long-reads sequencing data. Notably, these genes are associated with aging and longevity at both the gene level and at the network module level. The expression of these genes oscillates in response to caloric restricted feeding regimens, which have been demonstrated to promote longevity. In addition, aging and longevity genes are disrupted in various circadian disorders. Our study indicates that the modulation of circadian-affected tissue specificity is essential for understanding the circadian mechanisms that regulate aging and longevity at the genomic level.

## Abstract (web summary)

A multi-omics study on circadian rhythms in mice suggests that ~35% of tissue-specific genes may be influenced by circadian regulation, and these genes are linked to aging and longevity.

## Introdution

Tissue specificity refers to the unique genes expressed in different tissues. Many heritable diseases are tissue-specific, including aging-associated diseases^1^. The process of aging has been conceptualized as the greatest risk factor for diseases^2^, and this process is heterogeneous across different tissues^3^. Tissue-specific transcriptomic signatures have been reported in aging organs from human to *Caenorhabditis elegans*^4–7^. Recent studies have indicated that tissue specificity is lost during aging, leading to the phenomenon of coordinated global aging behavior across tissues^7–9^.

Circadian regulation has been shown to influence aging and longevity^10–12^. The circadian regulatory process involves several proteins that comprise a negative autoregulatory feedback loop^13,14^. In mice, the PER and CRY proteins are active in the late evening or at night, while the BMAL1 and CLOCK protein are active during the daytime^15^. The CLOCK–BMAL1 complex promotes the transcription of Per and Cry through binding to E-boxes. In turn, PER and CRY dimerize and interact with BMAL1-CLOCK complex to repress their own transcription at night^14^. The deficiency of CLOCK or BMAL1 protein can decrease the lifespan of mice^16,17^, and aging can impact the amplitude and phase of circadian rhythms^18–20^.

Circadian disturbances such as jet leg, have a significant effect on older mice compared to adult mice^21^ and can lead to increased mortality^22^. Shift work increases blood pressure and the risk of cardiovascular disease^23^. Many genes related to longevity are regulated by circadian genes^24,25^. Circadian rhythmicity is known to weaken with age, characterized by a decrease in the circadian amplitude, an advance or delay of the circadian phase, and a disturbances in cell and tissue synchronization^26–28^. Studies have shown that controlled feeding regimens can reset circadian rhythms and improve health^29,30^ and phase-aligned feeding activities can effectively delay aging and promote longevity^31^.

Tissue specificity can also be regulated by the circadian clock as well^32–36^ Each of the core circadian transcriptional factors binds to thousands of target genes across the genome^14,37,38^, coordinating the temporal functional organization of different organs^39,40^. However, The target genes of these circadian regulatory proteins vary in different organs in mice, as well as in other species like baboon^35,36,41–43^, owing to factors such as tissue-specific DNA binding events mediated by the transcription factor BMAL1^40,44^ and tissue-specific chromatin activities^45,46^. Meanwhile, the binding targets of other core clock transcription factors, such as CLOCK, PERs, CRYs, also differ tremendously^14^. Although the suprachiasmatic nucleus (SCN) is understood as the principal circadian pacemaker, the circadian regulation system is present in almost all tissues and cells^47,48^, suggesting it is involved in the regulation of tissue-specificity as a common molecular mechanism.

Although the relationship between tissue specificity and circadian regulation is well-established, the molecular mechanisms that underlie this connection remain elusive. In the present study, we defined tissue specificity as the differential transcriptomic signatures identified in a given tissue compared to other tissues. To quantify the relationship between circadian clock and tissue specificity, we conducted a large-scale sequencing of multiple peripheral circadian tissues (forebrain, cerebellum, liver, kidney) in both normal and sleep disrupted conditions. We firstly accessed whether tissue specificity and circadian oscillation are linked in both normal and sleep deprivation conditions. We then investigated the temporal organization of tissue-specific genes in liver and kidney across 24 h and identified distinct classes of tissue-specific genes that are affected to varying degrees by circadian rhythms. Based on these classes, we found that tissue-specific genes affected by circadian clock are involved in metabolism-related functions, enriched in specific cell types, and highly enriched certain co-expression modules. Finally, we explored the property of circadian-affected tissue-specific genes, and revealed that they were linked to aging and longevity.

## Results

### The circadian rhythmicity of gene expression is highly correlated with tissue-specificity

**Fig. 1:**
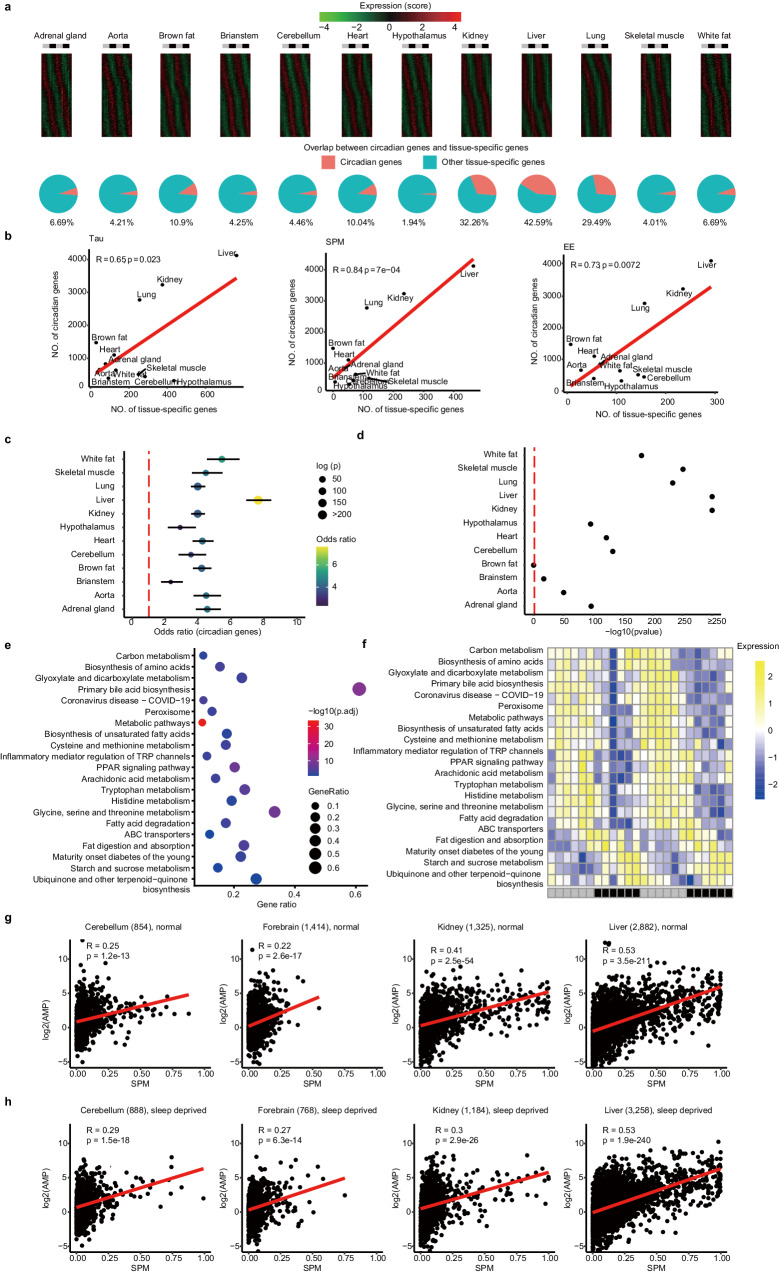
Correlation between tissue specificity and circadian rhythmicity across mouse organs. **a** The heatmaps display the tissue-specificity (measured by SPM) of circadian genes, and the pie charts show the proportion of them in tissue-specific genes (top 10% SPM score). R function pheatmap with the parameter scale = row was used to display the SPM changes of circadian genes. **b** The number of tissue-specific genes was positively correlated with the number of circadian genes in the 12 examined organs, as seen with tissue-specificity indicators including Tau, EE, and SPM. **c** Enrichment analysis of circadian Tau genes and rhythmically expressed genes. JTK_CYCLE was used to analyze Tau values of genes at different time points, and genes with Benjamini-Hochberg adjusted P < 0.05 were considered circadian Tau genes. Tissue circadian genes from the mouse circadian transcriptome atlas were also analyzed by JTK_CYCLE. **d** Enrichment analysis of tissue-specific genes (SPM) and tissue-specific genes (Tau). Tissue-specific genes were identified using the SPM value and Tau value calculated from the average gene expression in the mouse circadian transcriptome atlas and a cutoff value > 0.8. Tissue specific genes (Tau) is classified according to the tissue with the highest gene expression. **e** Enrichment analysis of biological processes of tissue-specific genes across circadian time in the liver. Both odds ratio and significance level (p) are indicated. **f** The heatmaps display the expression of enriched biological pathways across circadian time in the liver. **g** Scatter plots showing the relationship between the amplitudes of circadian genes and tissue specificity under normal conditions. In all four organs, tissue-specificity indicators (such as SPM) from the mouse circadian transcriptome atlas are significantly positively correlated with the amplitude of gene expression of the transcriptome (AMP), even in sleep deprivation conditions. Each dot represents a gene, and the Pearson correlation coefficient and its significance level (P) are displayed in each plot. **h** Scatter plots showing the relationship between the amplitudes of circadian genes and tissue specificity under sleep deprivation conditions.

To investigate the relationship between circadian rhythm and tissue specificity at the transcriptomic level, we explored the mouse circadian atlas^35^. We employed three data analysis methods — the tau index (Tau), specificity measure (SPM) and expression enrichment (EE) — to calculate the tissue specificity of genes expressed in 12 organs. We paid particular attention to how the expression of specific genes varied across different tissues over the 24 h circadian cycle. For each of the 12 organs examined, gene’s tissue specificity score oscillates across different time points (Fig. 1a). A significant positive correlation was detected between the number of tissue-specific genes and circadian genes (R = 0.65, R = 0.84 and 0.73, Fig. 1b, Pearson correlation). Liver and kidney, which were shown to have a higher number of cycling genes compared to other 10 organs, also had the most abundant tissue-specific genes (235 and 465 genes, respectively, compared to 7–119 genes in other organs).

Our analysis revealed that the tissue specificity of the identified genes exhibited temporal oscillations at different time points. We utilized JTK_CYCLE to calculate the circadian of gene expression in mouse 12 tissues (Jonckheere-Terpstra-Kendall test, q-value < 0.05). Notably, we found that the genes whose Tau values showed temporal oscillations were significantly enriched with the rhythmic genes of the tissue, with odds ratios greater than 3 and P values less than 2.2 x 10^−6^ for all tissues analyzed (Fig. 1c). Consistent results were obtained using another two-alternative gene-tissue specificity calculation methods, SPM and EE. In fact, the tissue specificities defined by the three methods are highly similar to each other (R > 0.8, Fig. 1d and Fig. S1). At the pathway level, these tissue specific genes are enriched in metabolic functional pathways (Fig. 1e), which also exhibited rhythmic oscillation signals throughout the day (Fig. 1f). So circadian regulation and tissue specificity are highly correlated throughout the body. Similarly, we analyzed the functional differences between tissues based on circadian genes. Specifically, we examined the top 10 biological processes of Gene Ontology (GO) annotation associated with circadian genes from the mouse circadian atlas. Our analysis revealed that circadian genes exhibit functional diversity, with significant associations to metabolism and biological rhythms (Fig. S2). Furthermore, we employed the Weighted Gene Co-expression Network Analysis (WGCNA) to explore overall gene expression patterns. This network was partitioned into several modules (Fig. S3a), and we identified those modules enriched with circadian genes. Notably, the liver and kidney exhibited the highest enrichment in circadian gene modules (Fig. S3b, c).

Circadian disturbances such as acute sleep deprivation have been shown to induce tissue-specific transcription and DNA methylation of human core clock genes^49^. To explore how circadian disturbance affects the relationship between circadian rhythm and tissue specificity, we conducted RNA-sequencing on 96 samples from both normal and sleep-deprived (circadian disrupted) mouse organs, including the liver, kidney, forebrain, and cerebellum. These organs were selected due to their previous demonstration of containing robust circadian expressed transcripts, and the forebrain and cerebellum are recognized as top influenced tissues by the circadian systems within the brain^35^. For sleep-deprived samples, mice were subjected to a 10 h sleep deprivation, as severe sleep deprivation can affect the transcriptome of mouse tissues (Maret et al. 2007). We collected two replicates every 4 hours from male mice, under constant darkness. We chose to sequence the samples at high depth, with an average of 54 million reads sequenced per sample. The reads were aligned to the mouse genome GRCm38 using HISAT2, and the transcription information for each annotated genomic region was estimated, facilitating downstream analysis^50^. This allowed us to access the expression signature for both protein-coding and noncoding transcripts. We detected a total of 18,531 transcripts with abundant transcriptional signatures, expressed in all samples, which were used for downstream analysis. To validate the accuracy of the sleep deprivation experiment, we analyzed rhythmic genes in each tissue. The findings revealed varied degrees of alteration in the expression of rhythmic transcriptomes and key circadian genes (Fig. S4).

Next, we analyzed the relationship between rhythmic expression signals in the four organs and the tissue-specific signals of the genes as measured by the specificity measure (SPM) from the mouse circadian transcriptome atlas. Although sleep deprivation altered the pattern of gene oscillations, we observed that the correlation between circadian amplitude (AMP) and tissue specificity of rhythmically expressed genes remained significant in both control and sleep-deprived conditions (Fig. 1g, h), particularly in the liver. We note that similar results were obtained when expressed genes were defined using different cutoffs (see methods). Importantly, when comparing the tissue specificity of rhythmically expressed genes with that of non-rhythmically expressed genes in calorie-restricted feeding states, we observed a significant difference. In both adult and old mice, the tissue specificity of rhythmically expressed genes was markedly greater than that of non-rhythmically expressed genes (Fig. S5). This finding suggests that the rhythmic expression of genes may play a crucial role in coordinating tissue-specific responses to calorie-restricted feeding.

### Both differentially expressed protein-coding and noncoding genes are temporally organized

To quantify the relationship between circadian regulation and tissue specificity, we focused on the circadian transcriptome of the liver, utilizing kidney as a reference organ, as these two peripheral organs contain the most robust circadian transcription patterns^35^. We profiled their 24 h circadian transcriptomes with four replicates collected every four hours under constant darkness, to confidently access their differentially expressed transcripts for each time point. More replicates allow us to identify the cycling genes more robustly and enable a more sophisticated computational analysis. For the protein-coding genes expressed in both organs, 3310 transcripts in the liver and 1125 transcripts in the kidney were finally identified as rhythmically expressed protein-coding genes (Jonckheere-Terpstra-Kendall test^51^, BH.Q adjusted P < 0.05).

We then identified the differentially expressed genes (DEGs) between the two organs to define tissue specificity, using both DESeq2^52^ and edgeR^53^ algorithms, with an adjusted p < 0.05 and greater than 2-fold change used as cutoffs. In total, 9,898 DEGs were identified between liver and kidney for the 6 time points. Strikingly, only 44% (4,454 genes) of these DEGs were defined as DEGs across all 6 time points, suggesting that most DEGs are time-dependent. To further assess the prevalence of common DEGs, we conducted a permutation test across all the samples. On average, 47% of genes were found to be differentially expressed at all time points under randomization experiments. Therefore, the observed value of 44% is slightly lower than expected by chance. These differences of gene expression profiles between liver and kidney reflect the temporal organization of the transcriptome.

**Fig. 2:**
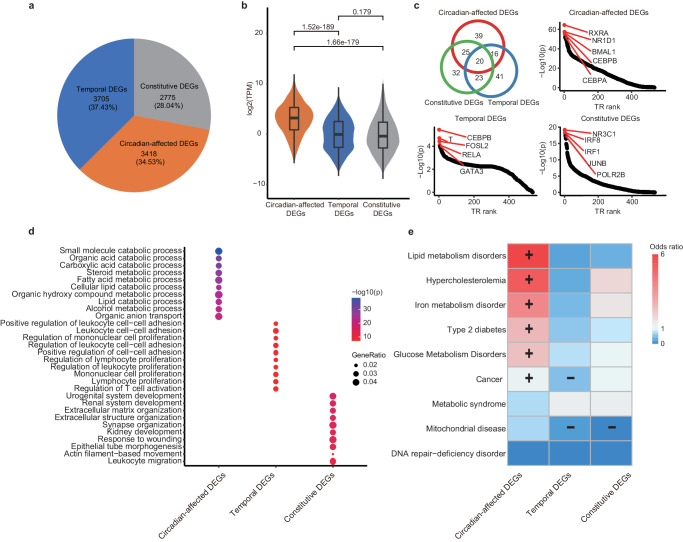
Temporal organization of differentially expressed genes (DEGs) between liver and kidney. **a** The pie chart displays the proportion and number of three classes of differentially expressed genes (DEGs). Among all DEGs, 35% are circadian-affected, 28% are constitutive, and 35% are temporal DEGs. **b** Expression abundance comparison among the three classes of DEGs. P-values were determined by Wilcoxon rank sum test. Error bars are the 95% confidence interval, the bottom and top of the box are the 25th and 75th percentiles in boxplot. **c** Differential transcription factors (TFs) potentially binding to distinct DEGs. Top TFs are indicated in each panel. The Venn diagram shows the number of TFs identified in overlap. **d** Functional annotation of the three DEG classes. Circadian-affected DEGs are enriched in metabolic functions, while other DEGs are associated with distinct biological pathways. Both enrichment odds ratio and significant level (P) are displayed. **e** Enrichment analysis of metabolic diseases for circadian-affected, constitutive, and temporal DEGs. + indicates significantly overrepresented, and - indicates significantly underrepresented. Different colors indicate odds ratios.

To assess the effect of circadian regulation on these DEGs, we further classified them into genes that are potentially affected by the circadian regulation and those that are not. The first category, circadian-affected DEGs, includes genes that are cyclic expressed in either liver or kidney with a 24-hour period, as well as genes that could be bound by the core circadian transcriptional factors (BMAL1, CLOCK, PERs, CRYs)^14^. The remaining DEGs can be divided into two categories: 1) constitutive DEGs, which are differentially expressed across all time points but are not affected by circadian regulation, and 2) temporal DEGs, which are differentially expressed in some time points but are not circadian-affected DEGs. We discovered that 35% (3,418) of DEGs are circadian-affected DEGs, while only 28% (2,775) of them are constitutive DEGs, demonstrating a larger contribution of circadian regulation to DEGs than expected (Fig. 2a and Supplementary Data 1).

Noncoding RNAs are transcribed widely throughout the genome, and exhibit oscillation signatures and high tissue-specificity. Long noncoding RNAs (lncRNAs) are a particularly noteworthy class, but their regulatory role in circadian rhythms remains incompletely understood^54^. We examined if and to what extend differentially expressed noncoding genes are temporally organized. We identified 161 rhythmic lncRNA transcripts among the 2273 expressed lncRNAs in liver and kidney. The proportion of rhythmic lncRNAs (7.1%) is lower than that of protein-coding genes (24.6%), consistent with previous observations^35^. We focused on two classes of non-coding RNAs interested: bi-promoter transcripts and antisense transcripts (Pelechano and Steinmetz, 2013). We estimated their differential expression signatures and identified 523 differentially expressed antisense transcripts and 181 differentially expressed bidirectional transcripts. In both categories, the proportion of constitutive differentially expressed noncoding transcripts is less than one-third of all differentially expressed noncoding transcripts (Fig. S6a, S6b). Thus, the majority of tissue specificity defined by noncoding RNAs also follow a time dependent manner.

The phase of rhythmical lncRNAs is close to that of their neighboring protein-coding genes, with the majority having phase differences of less than four hours (R = 0.42, P = 0.00048, Fig. S6c). This suggests that the oscillation of lncRNAs is coordinated with that of neighboring protein-coding genes, possibly because of chromatin remodeling. This is also evidenced by the observation that the location of cycling non-coding transcripts is co-clustered with cycling coding transcripts on the chromosome (Fig. S6d).

### Circadian-affected DEGs exhibit distinct functions compared to other DEGs

We discovered several key differences between circadian-affected DEGs and other DEGs. Circadian-affected DEGs typically have elevated transcription levels than other two groups of DEGs (median TPM expression level = 9.39 vs. 0.77, P < 2.2 x 10^−16^, Fig. 2b), but no differences in expression level were detected between constitutive DEGs and temporal DEGs (median TPM expression level = 0.77 vs. 0.98, P = 0.18, Fig. 2b). Moreover, differences in the expression levels of the three types of DEGs were observed to persist in the calorie-restricted mice, as illustrated in Figure S7a. Similar to the protein-coding genes, circadian affected DE noncoding genes also have higher expression level than other DE noncoding transcripts (median TPM expression level = 0.85 vs. 0.42, P = 1.7 x 10^−08^, Figure S6e). We also found that these three groups of genes are regulated by different transcription factors (Fig. 2c), with only 20 common TFs shared among the top 100 TFs (Supplementary Data 2). This implies a significant difference in the transcriptional regulatory structure of the three groups of genes. Circadian-affected DEGs were predicted to be regulated by a various circadian core clocks, including *RXRA*, *NR1D1*, *BMAL1*, and transcription factors such as *CEBPA* and *CEBPB*^55^, which are involved in liver metabolic processes. *CEBPB* is also the top regulator of temporal DEGs, which are also controlled by some general transcription factors involved in cell growth and replication, such as *FOSL2* and *RELA*. In contrast, *IRF1* and *IRF8* with immune function were involved in the regulation of constitutive DEGs^56–58^. Finally, in combination with recently released long-read sequencing data for the liver^59^, our analysis identified 59 Circadian-affected DEGs that are potentially fusion genes in cancer tissue, a significantly higher number than expected (R = 1.48, p = 0.0093, Fisher’s exact test). These fusion events tend to occur more frequently in the 5’ region than in the 3’ region, as shown in supplemental Fig. S8.

Interestingly, the three types of DEGs function in biological processes. GO functional enrichment results indicated that circadian-affected DEGs are extensively involved in liver-specific metabolic processes such as fatty acid and alcohol metabolism. Similar results were obtained by the KEGG analysis (Fig. S9). Temporal DEGs, on the other hand, play a significant role in immune cell proliferation and intercellular communication, while constitutive DEGs are linked to kidney functions such as urogenital system development. Furthermore, genes involved in cell movement, including leukocyte migration and actin filament-based movement, are also well-represented among constitutive DEGs and Temporal DEGs (Fig. 2d, Supplementary Data 3). The immune-related gene expression signatures observed in the temporal set were predominantly associated with immune cells, rather than the kidney or liver cells themselves. GO functional enrichment results of circadian genes in caloric restriction were found to be similar to those of circadian-affected DEGs, rather than other DEGs, as depicted in Figure S7b. Next, we investigated whether these three types of DEGs display different patterns by examining datasets for metabolism-related diseases and cancer^60^. The circadian-affected DEGs were highly enriched in metabolic disorders such as hypercholesterolemia and type 2 diabetes, consistent with their biological functions. Conversely, constitutive DEGs exhibited only weak enrichment in these diseases, while temporal DEGs did not show any clear trend (Fig. 2e, Supplementary Data 4). In summary, our findings demonstrate that the three DEGs have distinct transcriptional regulatory mechanisms, biological functions, and disease tendencies.

### Aging and longevity genes are enriched among the circadian-affected DEGs

**Fig. 3:**
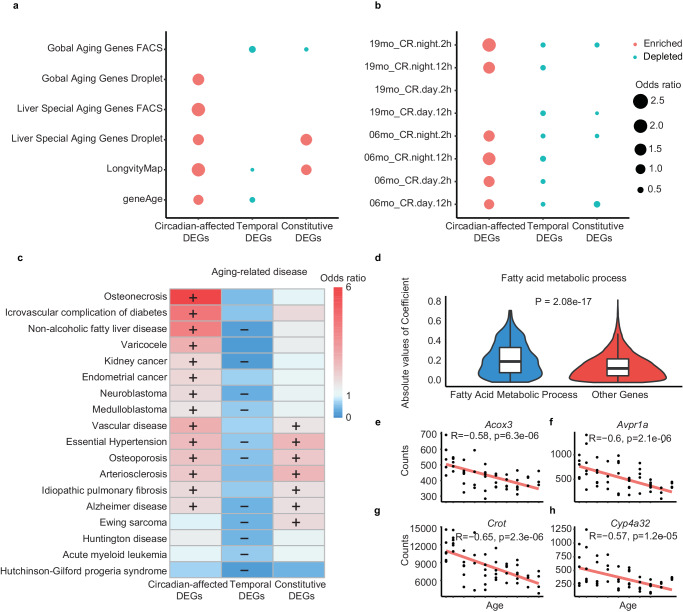
Relationship between circadian-affected DEGs and aging and longevity genes. **a** Enrichment analysis of three types of DEGs with aging and longevity genes. The “enriched” and “depleted” relationships are shown in red and blue, respectively. The odds ratio is indicated by the size of the circle. Fisher’s exact test was used for enrichment analysis, and the total gene set was the genes expressed in all samples. **b** Enrichment analysis of three types of DEGs with genes that oscillate in response to calorie and time restriction. Eight different intervention protocols were included: 06mo_CR.night.2 h, 06mo_CR.night.12 h, 06mo_CR.day.2 h, 06mo_CR.day.12 h, 19mo_CR.night.2 h, 19mo_CR.night.12 h, 19mo_CR.day.2 h, and 19mo_CR.day.12 h. Fisher’s exact test was used for enrichment analysis, and the total gene set was the genes expressed in all samples. **c** Functional enrichment analysis of aging-related diseases for circadian-affected DEGs, constitutive DEGs, and temporal DEGs. The enriched and depleted relationships are shown in red and blue, respectively. The odds ratio is illustrated by different colors. Fisher’s exact test was used for enrichment analysis, and the total gene set was the genes expressed in all samples. **d** Comparison of correlation coefficients between fatty acid metabolic genes and other genes with age. Error bars are the 95% confidence interval in boxplot. **e**–**h** Scatter plots showing the correlation between the expression levels of four circadian-affected DEGs (*Acox3*, *Avpr1a*, *Crot*, and *Cyp4a32*) and age. The Pearson correlation coefficient was used to calculate the correlation coefficient.

Aging and longevity processes have been shown to be closely linked with circadian regulation^3,10,12^. Therefore, we quantified the relationship between circadian-affected DEGs and aging & longevity genes at the genome-scale. Initially, we focused on the genes that exhibit aging-related expression changes in different cell types, which were identified using the Tabula Muris Senis single-cell dataset^61^. We used the Fisher’s exact test to analyze the association between aging and longevity genes with circadian-affected DEGs. The genes expressed in the sample were used as the total gene set, and the aging and longevity genes were required to be expressed in the sample. The circadian-affected DEGs are enriched in cell-type-specific aging changes when compared to the constitutive DEG and the temporal DEGs (Fig. 3a). This result was validated in two additional independent aging and longevity gene collections - LongvityMap database^62^, and GeneAge database (Fig. 3a, Supplementary Data 5).

As calorie restriction with aligned circadian phase can significantly promote longevity in mice^31^, we further focused on the genes that show circadian signals under both caloric and time restriction condition, where the oscillation is susceptible to these restrictions. We found that circadian-affected DEGs are significantly enriched in caloric & time restriction related circadian genes, whereas other DEGs are depleted in those conditions (Fig. 3b, Supplementary Data 6). Similar results were observed when we explored the reorganized liver circadian transcriptome of both young and old mice subjected to caloric restriction condition (Figure S10, P < 2.2 x 10^−16^ in all comparisons)^63^. Then we conducted a comparison of the fold change of genes among the three types of DEGs during caloric restriction (CR) and ad libitum feeding (AL). The findings revealed that only circadian-affected DEGs exhibited a significantly lower fold change compared to other genes (P = 0.0053), as illustrated in Figure S11a. These results suggest that tissues affected by circadian-driven specificity are involved in the aging process and are influenced by caloric restriction. Furthermore, we selected aging marker genes previously reported to be associated with oxidative stress effects and aging, and intersected them with liver tissue-specific genes to identify liver tissue-specific aging genes. Under caloric restriction, the expression of most of these genes exhibited a significant decrease in both mice, as depicted in Figure S11b. These results strongly indicate that the set of circadian affected DEGs is enriched for genes with functions potentially beneficial for lifespan extension^25,31^.

We found that Circadian-affected DEGs were enriched in various age-related diseases, as annotated by human genetic disease database^9^ (Fig. 3c). One of the top aging diseases that we identified was Nonalcoholic fatty liver disease, a common disease in elderly people^64^. Other diseases involving basal metabolic dysfunction, such as Osteonecrosis and cardiovascular complication of diabetes, were also significantly enriched in these DEGs. Interestingly, temporal DEGs exhibited the opposite trend, with diseases such as Arteriosclerosis being heavily depleted. By comparing the Spearman correlation coefficients between their expression level and age, it was found the correlation coefficients of fatty acid metabolic genes are significantly higher than those of other genes (Absolute values of Spearman: 0.21 vs. 0.14, *P* < 2.2 x 10^−16^, Fig. 3d). The expression of many genes in this function is anticorrelated with age, including *Acox3*, *Avpr1a*, *Crot* and *Cyp4a32* (Fig. 3e-h). The decreased expression of the four genes during aging may be related to weakened fatty liver metabolism and impaired liver function in elderly individuals.

### Circadian-affected DEGs are highly expressed in hepatocytes

**Fig. 4:**
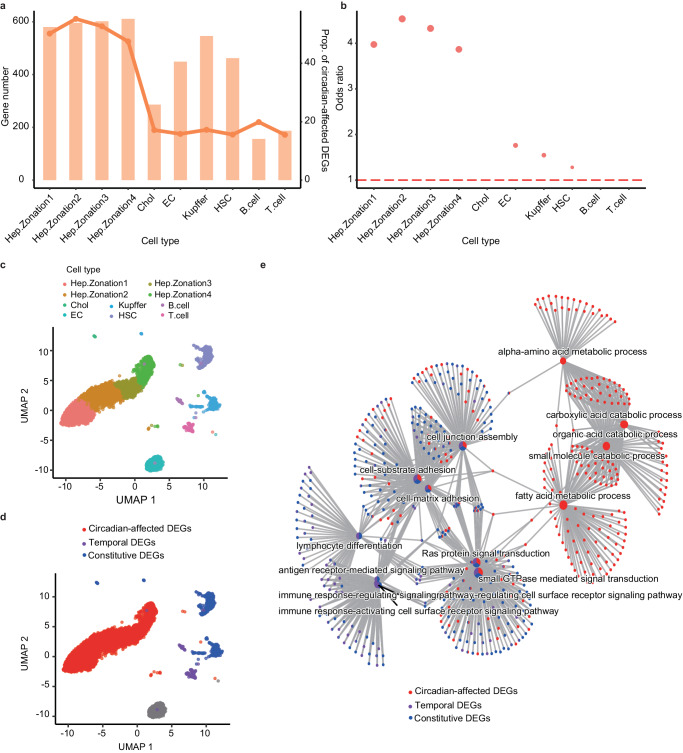
Cell-type specific analysis of circadian-affected DEGs. **a** The histogram shows the number of marker genes for different cell types, and the broken line represents the proportion of circadian-affected DEGs among the marker genes. **b** Enrichment of circadian-affected DEGs in marker genes for different cell types. **c** UMAP plot visualizing liver cell clusters based on single-cell transcriptomes. **d** UMAP plot visualizing the enrichment of the three classes of DEGs. The different colors indicate the cell types enriched by different classes of DEGs, while gray indicates no enrichment of any DEG. **e** Functional network annotation of the three classes of DEGs based on enriched cell type marker genes. The dots of different colors represent different classes of DEGs.

Tissue-specificity may arise from tissue-specific cell types, given that most genes specifically expressed in a tissue are enriched in certain cell types^65^. At the single-cell level, both hepatocyte and other cells in the liver are under the control of the circadian clock^66^. To investigate the cell-type origin of the circadian tissue specificity in the liver, we compared the cell-type marker genes in each cell population^66^. In total, 2,587 cell-type marker genes were identified in 10 cell types, ranging from 150 to 600 genes per cell type (Fig. 4a). In hepatocyte cells, ~40% of cell markers were circadian-affected DEGs, while in other cell types (*e.g*., Chol and EC), only ~20% cell markers were circadian-affected DEGs (Fig. 4a). Cell marker genes tend to be circadian-affected DEGs in 4 out of 10 liver cell types (*P* < 2.2 x 10^−16^, Fig. 4b). Through analysis in the Cistrome DB database combined with large-scale CHIP-Seq data, we discovered that hepatocyte cells markers potentially bind with circadian core transcription factors such as CLOCK, BMAL1, CRY1, CRY2, while other cell types markers are more divergent (Figure S12). In addition, the circadian genes under both caloric and time restriction condition are mostly enriched in the four major hepatocyte cells (Odds ratio > 3 for all of them, P < 2.2 x 10^−16^), indicating that hepatocytes actively participate in the circadian oscillation of gene expression under metabolic restructuring^55^.

The analysis of DEGs and cell marker genes revealed that the circadian-affected DEGs are enriched in hepatocyte cells (Hep Zone1, *P* < 2.2 x 10^−16^; Hep Zone2, *P* < 2.2 x 10^−16^; Hep Zone3, *P* < 2.2 x 10^−16^; Hep Zone4, *P* < 2.2 x 10^−16^), while temporal DEGs are enriched in immune cells (*P* < 0.05), and constitutive DEGs are enriched in other cell types (*P* < 0.01) (Fig. 4c, d). This suggests that immune cells and epithelial cells in the liver may contribute less to the overall tissue specificity. Similarly, the functional annotation of cell-type marker genes indicated that hepatocytes cell markers were involved in metabolic functions, such as fatty acids and amino acids metabolic process, and formed a single cluster, while genes for cell interactions and immune function were shared among cell types to varying degrees (Fig. 4e). These results reveal that the three types of DEGs are derived from different cell types dynamically, suggesting that different cell types contribute to different aspects of the temporal distribution of tissue-specific gene expression.

### Cycling co-expression modules are associated with aging

**Fig. 5:**
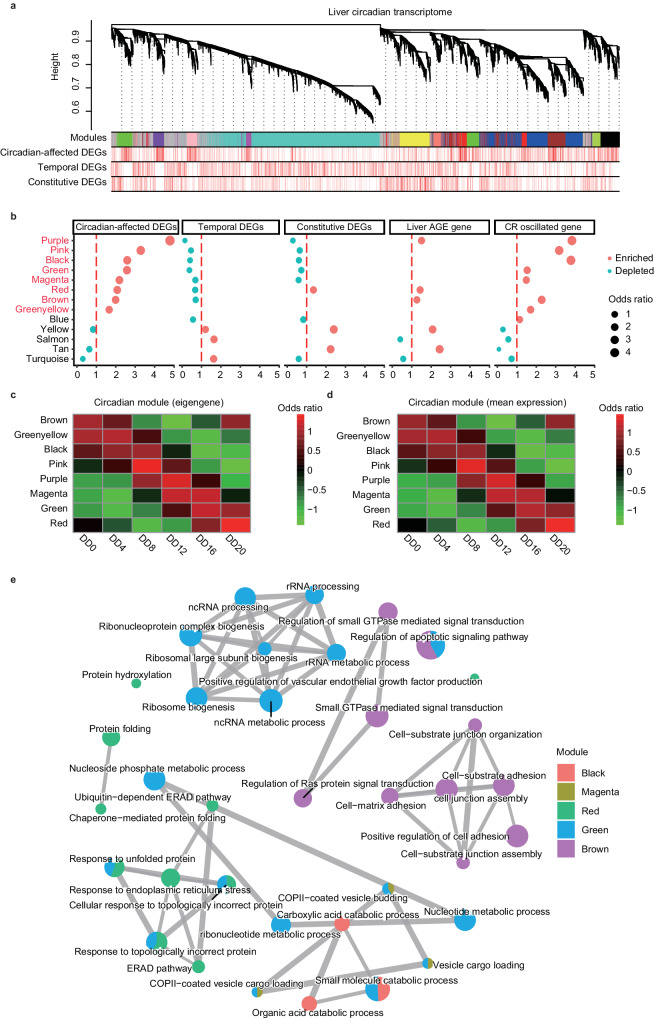
Weighted gene co-expression network analysis (WGCNA) of circadian-affected DEGs. **a** The co-expression network of liver samples visualized using a cluster diagram, with different types of DEGs assigned to the identified modules. **b** Identification of cycling modules with enrichment of circadian-affected DEGs, liver-specific aging genes, and rhythmic genes under caloric and time restriction conditions. The odds ratio is shown on the X-axis, and co-expression modules are shown on the Y-axis. Again, “enriched” is indicated by red dots, while “depleted” is indicated by blue dots. **c** Heatmap of the eight circadian modules ordered by circadian phase, showing module eigengene values. **d** Heatmap of the eight circadian modules ordered by circadian phase, showing averaged expression levels. **e** Functional annotation of the cycling modules, showing their involvement in processes such as response to protein folding, cell junction assembly, rRNA processing, rRNA metabolic processes, and small GTPase-mediated signal transduction.

To understand the modular properties of circadian-affected DEGs, we next conducted the weighted gene co-expression network analysis (WGCNA) on liver circadian transcriptomes across all 24 samples. We identified a total of 13 co-expression network modules, with module size ranging from 249 to 5466 genes (Fig. 5a, b, Supplementary Data 7). We then investigated the rhythmicity of eigengene expression in each module (Fig. 5c, d). Our findings were consistent with the previous report^67^, which demonstrated that a significant number of network modules display a rhythmic expression pattern. Specifically, eight out of the 13 modules are cycling modules (black, greenyellow, brown, green, pink, purple, magenta, and red, *P* < 0.001, BHQ method; Fig. 5b), indicating that the gene regulatory network changes dynamically throughout the day.

We then assessed the enrichment of three groups of DEGs in those modules. We found that all eight circadian-expressed modules were enriched with circadian-affected DGEs (Fig. 5b, c). Moreover, the distribution of temporal DEGs and constitutive DEGs in the module was opposite to circadian-affected DEGs, with these genes being significantly depleted in cycling module. Additionally, we found that among these cycling modules, the purple, brown and red modules were also enriched with liver-specific aging-related genes. Remarkably, 7 of all 8 cycling modules were highly associated with circadian genes under both caloric and time restriction condition (Fig. 5b), indicating the robustness of oscillations of network modules under restricted dietary conditions. This suggests the robustness of oscillations of network modules under different dietary conditions and that aging-related genes also show higher levels of circadian rhythm at the module level in the gene regulatory network.

Functional annotation of these circadian modules uncovered that they were mainly involved in response to protein folding (*P* < 1.99 x 10^−12^), cell junction assembly (*P* < 6.50 x 10^−^^07^), rRNA processing (*P* < 2.2 x 10^−^^10^), rRNA metabolic process (P < 1.37 x 10^−10^) and small GTPase mediated signal transduction (P < 7.08 x 10^−^^08^) (Fig. 5e), which have been reported to be associated with aging and longevity in previous studies^68^. In summary, the results above indicate that the differential genes in different tissues are aggregated in the gene expression network in the form of modules, which show cyclic expression together and are involved in the liver aging process.

### Aging genes are more disrupted in circadian misalignment environments compared with other genes

Circadian disruption is a deleterious condition that can lead to various diseases such as diabetes and cancer. We investigated whether the regulation of aging related genes is more susceptible to disturbance than other genes in the cycling transcriptome in circadian misalignment environments. To address this impact, we collected data on different circadian disruption conditions, such as sleep deprivation and an inverted diet. For both datasets, we utilized JTK_CYCLE to calculated the oscillation amplitude in the experimental and control groups and used the amplitude change between these groups to assess effect size for the examined gene sets. Briefly, we examined six sets of aging-related genes—obtained from the LongvityMap &GeneAge database—and the aging genes obtained from the Tabula Muris Senis single-cell dataset.

**Fig. 6:**
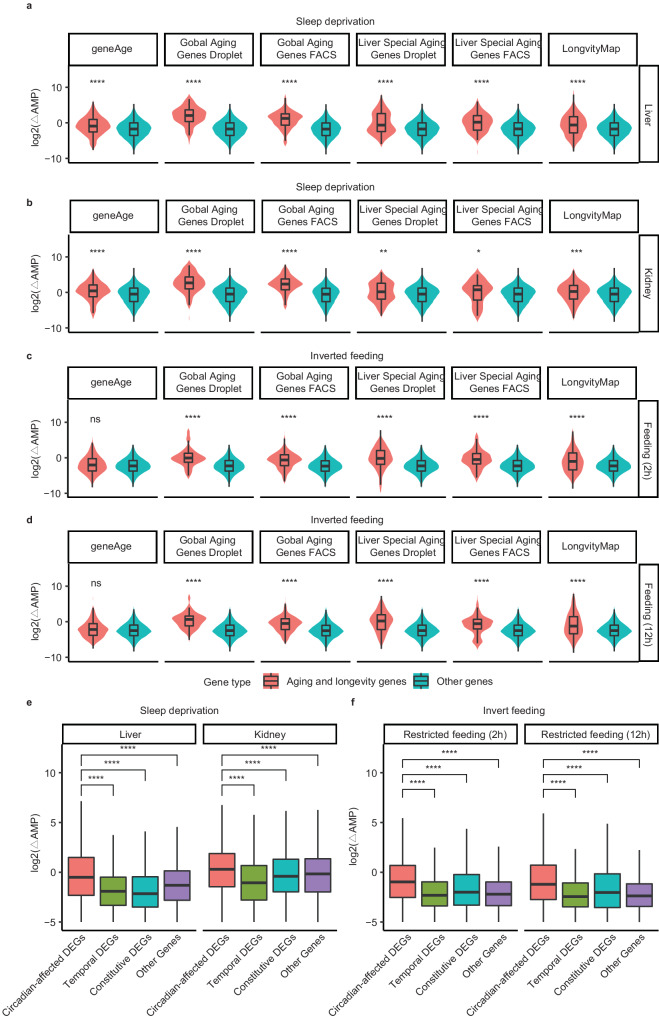
Genes related to aging are disturbed in sleep and diet disruption conditions. **a** A violin plot is used to show the change in circadian amplitude (AMP) of aging and longevity genes in the liver in comparison to other genes during 10 h sleep deprivation. All 6 aging and longevity gene lists are included in the plot. **b** The change in circadian amplitude (AMP) of aging and longevity genes in the kidney is displayed in a violin plot, compared to other genes during 10 h sleep deprivation. All 6 aging and longevity gene lists are included in the plot. **c** A violin plot to compare the change in circadian amplitude (AMP) of aging and longevity genes with that of other genes during a 2 h restricted inverted feeding condition. **d** A violin plot is used to compare the change in circadian amplitude (AMP) of aging and longevity genes with that of other genes during a 12 h restricted inverted feeding condition. **e** A boxplot to compare the changes in circadian AMP for circadian-affected DEGs, constitutive DEGs, temporal DEGs, and other genes during sleep deprivation. **f** A boxplot to compare the changes in circadian AMP for circadian-affected DEGs, constitutive DEGs, temporal DEGs, and other genes during inverted food intake condition. Both conditions exhibit similar trends. Statistical significance is denoted by ***P < 0.001; **P < 0.01; *P < 0.05; and n.s. for non-significant differences. Error bars are the 95% confidence interval, the bottom and top of the box are the 25th and 75th percentiles in boxplot.

As displayed in Fig. 6a, the cycling amplitude of aging-related genes exhibits a significantly larger change than other genes under 10 h acute sleep deprivation condition in the liver (total amplitude difference: 1.14 vs 0.30, *P* < 4.5 x 10^−^^05^, Fig. 6a). Similar results were observed when we performed downsampling to equalize the number of genes between groups (Fig. S13) and a similar trend was also observed in the kidney (total amplitude difference: 2.41 vs 0.71, *P* < 0.05, Fig. 6b). Moreover, we compared essential genes and found that they are less affected by sleep deprivation, exhibiting more robust expression (Fig. S14). These results suggest that the circadian expression of aging-related genes in peripheral organs is susceptible to sleep behavior interference, meaning that sleep deprivation can exert pressure on the rhythmic expression of the circadian-affected DEGs in the aging process.

In the context of a restricted diet that is known to improve the circadian rhythm of mouse behavior^69–71^, our findings reveal a similar pattern: the circadian amplitude of aging gene expression responds more dramatically to this intervention (total amplitude difference: 0.55 vs 0.19, *P* < 0.05, Fig. 6c, d). This suggests that the change may not be limited to only sleep or diet, but rather a broader molecular mechanism that underlies both and plays a role in the amplitude sensitivity of aging genes. In addition, circadian-affected DEGs had larger amplitude changes in the two organs than the other DEGs and than the non-DEGs (DEGs amplitude difference: 0.81 vs 0.32 vs 0.25, *P* < 2.2 x 10^−16^, Fig. 6e). This is consistent with the results of aging genes, supporting the close relationship between aging genes and DEG. We also noted similar observations in the inverted feeding condition (2 h, or 12 h inverse in adult mice, Fig. 6f)^31^ in the diet disruption condition, which was also validated by analyzing the recently released inverse-feeding peripheral tissues transcriptome dataset^72^ (Fig. S15). Consequently, circadian disruption is closely linked with circadian-affected DEGs and aging genes.

## Discussion

In this study, we explored the temporal distribution of tissue-specific genes and their relationship with circadian regulation. We found that both protein-coding and noncoding genes exhibited strong tissue-specific expression patterns that varied over time. Notably, the tissue specificity affected by circadian regulation differed from other tissue specificities in several important ways, such as high expression level and enriched functional pathways, suggesting that the circadian clock plays a unique role in modulating their gene expression. Furthermore, our analysis revealed that circadian-affected DEGs were enriched in aging and longevity-related pathways, with significant associations to metabolism. Specifically, we observed that these genes were predominantly expressed in hepatocytes within the liver, suggesting that these cells are required in regulating circadian rhythms and metabolic processes. We also found that the circadian co-expression modules containing these circadian-affected DEGs overlapped with the genes that oscillate under caloric and time restriction conditions. This suggests that these genes may play a crucial role in mediating the effects of these interventions on longevity and aging. Given that circadian phase-aligned interventions have been shown to promote longevity^31^, our findings suggest that these circadian-affected tissue-specific genes may represent a profound mechanism for extending lifespan at the molecular level.

Aging is a multifaceted process characterized by tissue-specific changes. During aging, the mammalian tissue transcriptome tends to exhibit similar expression levels, especially in genes specific to certain tissues, which can lead to the loss of tissue identity^7–9^. Additionally, in old mice, the number of genes that exhibit circadian expression patterns decreases, along with a decrease in the amplitude of circadian rhythms^31^, indicating a weakened circadian control of tissue specificity. We found that circadian-affected DEGs were highly enriched in aging-related genes, and also associated with several age-related diseases. Specifically, we investigated Nonalcoholic Fatty Liver Disease, a common condition in elderly individuals, and found that the expression levels of several circadian-affected DEGs were significantly negatively correlated with age, suggesting a possible association with the decline in liver metabolism related to fatty liver and the loss of liver tissue specificity during aging. These findings support that alterations in the circadian system can contribute to the loss of tissue identity during aging.

While previous studies have demonstrated that the size of liver cells and liver mass exhibit circadian oscillations in response to feeding-fasting cycles^73^, and different hepatocyte cell types are affected by the circadian rhythm to varying degrees^66^, the specific cell types that benefit from time-restricted eating remain unknown. Our analysis revealed that the main cell type that benefits from time-restricted eating may be hepatocyte cells, which is consistent with the enrichment analysis results from genes rhythmically expressed under caloric and time-restriction conditions. Interestingly, we also observed slight over-enrichment in other cell types such as EC, Kupffer, and HSC cells, suggesting that the full beneficial cells are complex and involve multiple cell types. Future studies using single-cell sequencing technologies can provide a deeper understanding of the cell-specific contributions of time-restricted eating. Moreover, it would be interesting to identify the molecular pathways and signaling molecules that are involved in this process.

Circadian disturbances have been implicated in a range of health issues, from metabolic disorders to neurodegenerative diseases. In fact, numerous studies have demonstrated a strong association between circadian disruption and various complex diseases, including accelerated aging, cancer, and cardiovascular disease^23,29^. In mice with sleep disturbances, the expression of the PER2 gene in peripheral tissues is altered, resulting in disrupted circadian clock^74^. Similarly, in individuals with inverted timing of food intake, the phase of a large number of genes is changed^72^. Interestingly, for both disturbances, our data indicate that the extents of the changes in circadian amplitude were more pronounced in aging and longevity genes compared to other genes in both of these disturbances. A greater change in circadian amplitude was observed in circadian-affected DEGs compared to other genes. Similarly, the circadian-affected DEGs exhibited the greatest changes in the liver during the aging process. This suggests that the impact of circadian misalignments on aging may be mediated through the effect on circadian-influenced tissue-specific genes.

While our study sheds light on the link between circadian-controlled tissue specificity and aging, there are several limitations to consider. Firstly, we did not differentiate between the three categories of differentially expressed genes (DEGs) in aging tissues, but instead relied on previously published aging circadian transcriptomes to connect circadian-affected DEGs with aging. This method may be restricted since the three classes of DEGs reflect various aspects of tissue specificity. Future studies should distinguish between the three classes of DEGs and explore their relationship with circadian regulation in aging tissues. Secondly, although we investigated several organs in this study, the aging process affects nearly all organs. Therefore, more tissues should be examined in future studies to investigate the role of tissue-specific circadian outputs in the aging process. This could provide a more comprehensive understanding of the intricate relationship between circadian rhythm and aging. Thirdly, our analysis was based on single cells from adult tissues, which may not fully represent the cellular composition of different cell types in aging tissues. Finally, while we have tried our best to minimize the introduction of systematic biases during the integration of multimodal data, it is essential to acknowledge that additional experimental evidence is required to further enhance the robustness of our findings. Despite these limitations, our study provides valuable insights into the connection between circadian-regulated tissue specificity and aging.

Together, our results suggest that the circadian-affected tissue-specificity plays an essential role in the aging process. The enrichment of these genes involved in calorie and time restriction that can delay aging and extend lifespan underscores the importance of tissue-specific circadian regulation in aging and longevity. Our study provides a deeper understanding of the complex relationship between circadian rhythms, tissue-specificity and aging, highlighting the potential for targeted interventions to improve healthspan and lifespan.

## Methods

### Animal preparation and sample collection

The C57/BL6 male mice used in this study were raised in a laboratory with a 12 h light-dark cycle (lights on at 8 a.m. and off at 8 p.m.). After entrainment to this cycle for five days, the mice were transferred to constant darkness (DD) and sacrificed 24 h later. We collected four replicates of liver and kidney every four hours throughout the day, while keeping the mice in constant darkness (DD) and providing free access to food and water. We collected liver and kidney samples from the same mice at each sampling time and immediately put them in liquid nitrogen. All animal experiments were approved by the Animal Care and Use Committee of the Shanghai Institute of Nutrition and Health and were conducted in accordance with institutional guidelines. We have complied with all relevant ethical regulations for animal use.

### Sleep deprivation experiment

The sleep deprivation experiment followed previously reported protocols^75^. We utilized male C57BL/6 mice, aged between 6 and 8 weeks, which were kept in an environment with a 12 h light/dark cycle. Water and food were provided ad libitum. Mice were randomly assigned to either the experimental or control group. The experimental group was subjected to a sleep deprivation instrument, which involved forced locomotion through a slowly rotating drum (40 cm in diameter, 0.4 m per min) while having access to food and water. All mice in experimental group were placed in the sleep deprivation device for 10 h (6:00 AM–4:00 PM) during their normal resting phase while the control animals were undisturbed, because acute sleep deprivation, lasting between 6 and 10 h, can leads to noticeable alterations in mice’s circadian rhythms and significantly affects the rhythmic expression of numerous genes^75–77^. Then we collected two replicates of 4 peripheral circadian tissues (forebrain, cerebellum, liver, kidney) were obtained at next six circadian time (CT) points with an interval of 4 h (CT4, CT8, CT12, CT16, CT20, and CT24).

### RNA-sequencing experiment

We extracted total RNA from tissue samples using Trizol (Invitrogen). After separating the RNA, DNA, and protein layers with chloroform, we precipitated the RNA using a standard isopropanol/ethanol procedure. The resulting RNA pellet was washed, and then resuspended in RNase-free water. We assessed the quantity and quality of the total RNA using the Qubit RNA HS Assay (Life Technologies) and RNA 6000 Nano Assay on a Bioanalyzer 2100 (Agilent). For RNA-seq library construction, we followed the Illumina TruSeq Total RNA stranded protocol. We used 1 μg of total RNA and depleted rRNA using the ribo-zero rRNA gold removal kit. We then chemically fragmented the rRNA-depleted RNA for three minutes and prepared a paired-end 150-bp strand-specific RNA-seq library using the NEB Next Ultra II Directional RNA Library Prep Kit (NEB 7760), following the manual. We performed deep sequencing using the Illumina HiSeq X platform. All our new samples have passed RNA sequencing quality control (RNAseq QC). We deposited all raw sequencing data in the Gene Expression Omnibus for free access accession.

### RNA-sequencing data processing

We used Trim Galore to assess quality and remove adapters from the FastQ files (parameters: --phred33 --length 50 --stringency 3 --paired) (https://github.com/FelixKrueger/TrimGalore, v0.6.4). We aligned the reads to the mouse reference genome (GRCm38, downloaded from GENCODE) using HISAT2 (version 2.1.0)^50^ with parameters -t -p 8 --dta --rna-strandness RF. We extracted high-quality alignment results from BAM files using Samtools (version 1.9)^78^ with parameters view -b -q 60. After alignment, we calculated read counts using featureCounts (version v1.6.4)^79^ with parameters -T 16 -p -s 2. We then created a final list of genes that were expressed (TPM > 0) in all samples.

### Differential gene expression analysis

We used raw counts to calculate differentially expressed genes. Differential expression analysis was performed using both DESeq2 (version 1.26.0)^52^ and EdgeR (version 3.28.1)^53^ between liver and kidney at each time point. We considered a gene to be differentially expressed if the adjusted p-value was less than 0.05 in both DESeq2 and EdgeR, which was selected as the significance cutoff.

### Identification of circadian expressed genes

We used the Jonckheere-Terpstra-Kendall algorithm from the MetaCycle R package (version 1.20)^80^ to identify oscillating genes from the cycling transcriptome. The algorithm was run with TPM as input, and we considered only genes with a Benjamini-Hochberg adjusted p-value of less than 0.05 as confidently circadian expressed genes.

### Identification of genes that oscillate exclusively under both caloric and time restriction condition

To identify circadian genes under caloric restriction conditions, we used the method adopted in the corresponding articles of each dataset to identify circadian rhythm genes. We employed the JTK algorithm from the MetaCycle R package (version 1.20)^80^ with a p-value cutoff of 0.01, using data from Sato et al.^63^. We selected genes that showed rhythmic expression only under caloric restriction conditions as our gene list. To identify circadian genes that oscillate exclusively under both caloric and time-restricted conditions, we used data from Acosta-Rodríguez et al.^31^, and selected cycling genes that were significant in all three different algorithms (JTK_CYCLE, ARSER, and RAIN) with a p-value cutoff of 0.05 and FDR < 0.05. We selected our final gene sets by filtering genes that were rhythmically expressed exclusively under both caloric and time-restricted conditions, but not under ad libitum or CR-spread conditions.

### Estimation of tissue-specificity and tissue-specific gene

To evaluate tissue-specificity of the transcriptome, we calculated Tau for each gene, using the following formula^81^:

$${\rm {T}}=\frac{{\sum }_{i=1}^{n}(1-\bar{{x}_{i}})}{{\sum }_{i=1}^{n}{x}_{i}^{2}}{{{{{\rm{;}}}}}}\,\bar{{x}_{i}}=\frac{{x}_{i}}{{\max }_{1\le i\le n}({x}_{i})}$$

Where, x_i_ is the expression level of  a given gene  in  tissue  i and n  is  the  number  of  tissues in a given time point.

We used another tissue-specificity indicator, specifically the Specificity Measure (SPM), to evaluate tissue-specificity of the transcriptome. SPM was calculated for each gene using the following formula^82^:

$${{{{{\rm{SPM}}}}}}=\frac{{x}_{i}^{2}}{{\sum }_{i=1}^{n}{x}_{i}^{2}}$$

Tissue-specific gene was defined as genes with SPM in top 10% of all genes.

We also used an additional tissue-specificity indicator, expression enrichment (EE)^83^, to validate our main discoveries. The EE score was calculated as following:

$${{{{{\rm{EE}}}}}}=\frac{{x}_{i}}{{\Sigma }_{i=1}^{n}{x}_{i}* \,\frac{{s}_{i}}{{\Sigma }_{i=1}^{n}{s}_{i}}}=\frac{{\Sigma }_{i=1}^{n}{s}_{i}}{{s}_{i}}* \frac{{x}_{i}}{{\Sigma }_{i=1}^{n}{x}_{i}}$$

S_i_ summary of the expression of all genes in tissue i

### Gene co-expression network analysis

For the weighted gene co-expression network analysis (WGCNA)^84^, we used the rlog transformation in the DESeq2 package to standardize the original RNA-Seq count data, which has a similar variance stabilizing effect to the varianceStabilizingTransformation. We selected genes with a Median Absolute Deviation (MAD) > 0.1. We then constructed co-expression networks using the Bicor method to calculate expression similarities. The softpower parameter was chosen as 14, resulting in 13 co-expression modules containing 17,070 expressed genes. To determine whether a module was rhythmic, we analyzed both the module eigengene and the mean expression of the module using JTK_CYCLE. Modules with a Benjamini-Hochberg adjusted *P*-value < 0.05 were considered circadian modules.

### Transcriptional factor (TF) prediction

We used Landscape In Silico Deletion Analysis (LISA v2.2.5)^85^ to predict potential transcription factors. For each analysis, we input the top 500 genes with the highest fold-change among each type of differentially expressed genes (DEGs).

### Biological function enrichment analysis

We performed functional annotation of genes using ClusterProfiler (version 4.0)^86^. Each term was ranked according to its false discovery rate (FDR), and the significance threshold was set to < 0.05. Since many enriched terms had intersections, we clustered similar terms based on their semantic similarity using a method described by Wang et al.^87^.

### Gene sets for metabolism-related diseases

Metabolic-related diseases were identified based on a previous study^60^. Cancer-associated genes were obtained from experimental data reported in the cancer gene census or from significantly enriched driver genes included in the Cancer Genome Atlas (TCGA, https://portal.gdc.cancer.gov/). Disease genes associated with metabolism were extracted from the Human Gene Mutation Database (HGMD)^88^ by selecting gene sets with at least one or more disease-associated mutations.

### Gene sets for aging-related diseases

We obtained aging-related diseases from a previous report^9^. Related disease gene sets were obtained from the MalaCards Human Disease Database^89–91^. To identify the corresponding mouse orthologs, we used the Ensembl Biomart^92^ to convert the human genes.

### Single cell marker gene identification

Marker genes for peak-based clustering were identified using Seurat’s FindAllMarkers() function^93^ on the gene activity matrix with the following parameters: min.pct = 0.25 and logfc.threshold = 0.25.

### Calculating the phase correlations between circadian lncRNAs and protein-coding genes

We calculated the correlations of time-course data between circadian lncRNAs and neighboring protein coding genes by selecting only those lncRNA clusters and their nearby protein-coding genes that were circadian expressed. For each lncRNA cluster, we calculated Pearson’s correlation coefficients between the phases of circadian lncRNA transcripts and circadian protein-coding genes located within a 50 kb region. The median of these correlation coefficients was used as the final correlation coefficient between this lncRNA and adjacent protein-coding genes in this region.

### Statistics and reproducibility

All the analyses were performed in R (v4.0.2). Statistical significance was determined at Benjamini-Hochberg adjusted *P*-value < 0.05. Gene set enrichment analysis was performed using a two-sided Fisher’s exact test.

### Reporting summary

Further information on research design is available in the Nature Portfolio Reporting Summary linked to this article.

### Supplementary information

Supplementary Information

Description of Additional Supplementary Files

Supplementary Data 1

Supplementary Data 2

Supplementary Data 3

Supplementary Data 4

Supplementary Data 5

Supplementary Data 6

Supplementary Data 7

Reporting Summary

## Data Availability

All raw and processed sequencing data generated in this study have been submitted to the NCBI Gene Expression Omnibus (GEO; https://www.ncbi.nlm.nih.gov/geo/) under accession number GSE240693, which will be freely accessible upon the publication of the manuscript.

## Code availability

We used R for most of the analyses. All original code is available upon request.
