# Supplementary material for: Circadian-driven tissue specificity is constrained under caloric restricted feeding conditions
Source: Commun Biol. 2024 Jun 20;7:752. doi: 10.1038/s42003-024-06421-0 (PMC11190204; doi:10.1038/s42003-024-06421-0)
Supplement: Supplementary file 1 — Supplementary Information [file 42003_2024_6421_MOESM1_ESM.pdf]

Supplementary Information

Circadian-driven tissue specificity is constrained under caloric restricted feeding conditions

Renrui Chen[1,5], Ziang Zhang[1,5], Junjie Ma[1,5], Bing Liu[2,5], Zhengyun Huang[3], Ganlu Hu[4], Ju Huang[2], Ying Xu[3], Guang-Zhong Wang[1,*]

[1] CAS Key Laboratory of Computational Biology, Shanghai Institute of Nutrition and Health, University of Chinese Academy of Sciences, Chinese Academy of Sciences, Shanghai 200031, China

[2] Collaborative Innovation Center for Brain Science, Department of Anatomy and Physiology, Shanghai Jiao Tong University School of Medicine, Shanghai 200025, China

[3] Jiangsu Key Laboratory of Neuropsychiatric Diseases and Cambridge-Su Genomic Resource Center, Medical School of Soochow University, Suzhou, Jiangsu 215123, China

[4] Shanghai Institute for Advanced Immunochemical Studies, ShanghaiTech University, Shanghai, China

[5]These authors contributed equally

*Corresponding author: Tel: (0086) 021-54920578;

Email: guangzhong.wang@picb.ac.cn (G.W.)

This PDF file includes:

Supplementary Fig. 1 to 15

Supplementary Figure

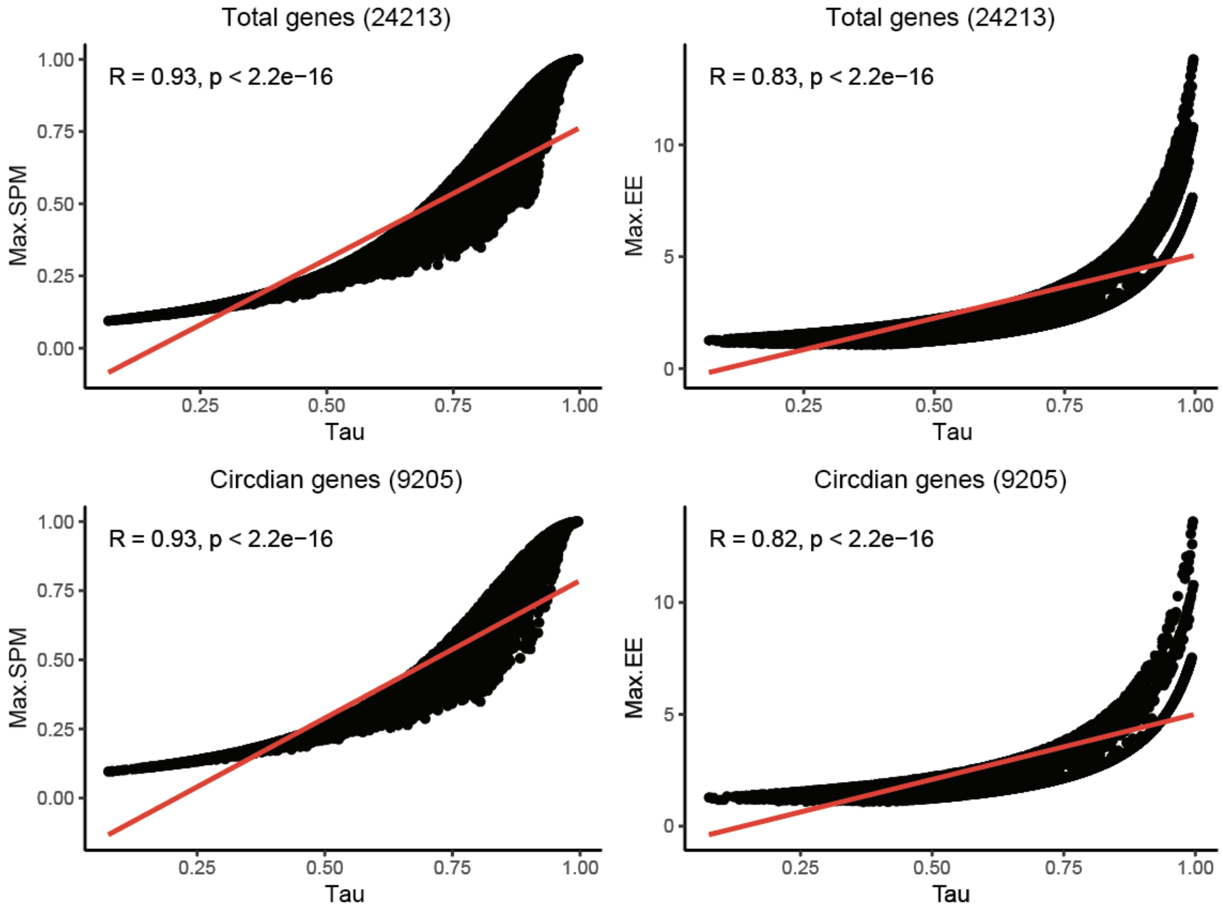

Figure S1. Correlation of different tissue specific value calculation methods. Scatter plot showing the relationship between calculated tissue-specific Tau values and tissue-specific SPM and EE values. The circadian genes was the union of circadian genes across mouse organs. Max.SPM and max.EE refers to the maximum value of genes between mouse organs.

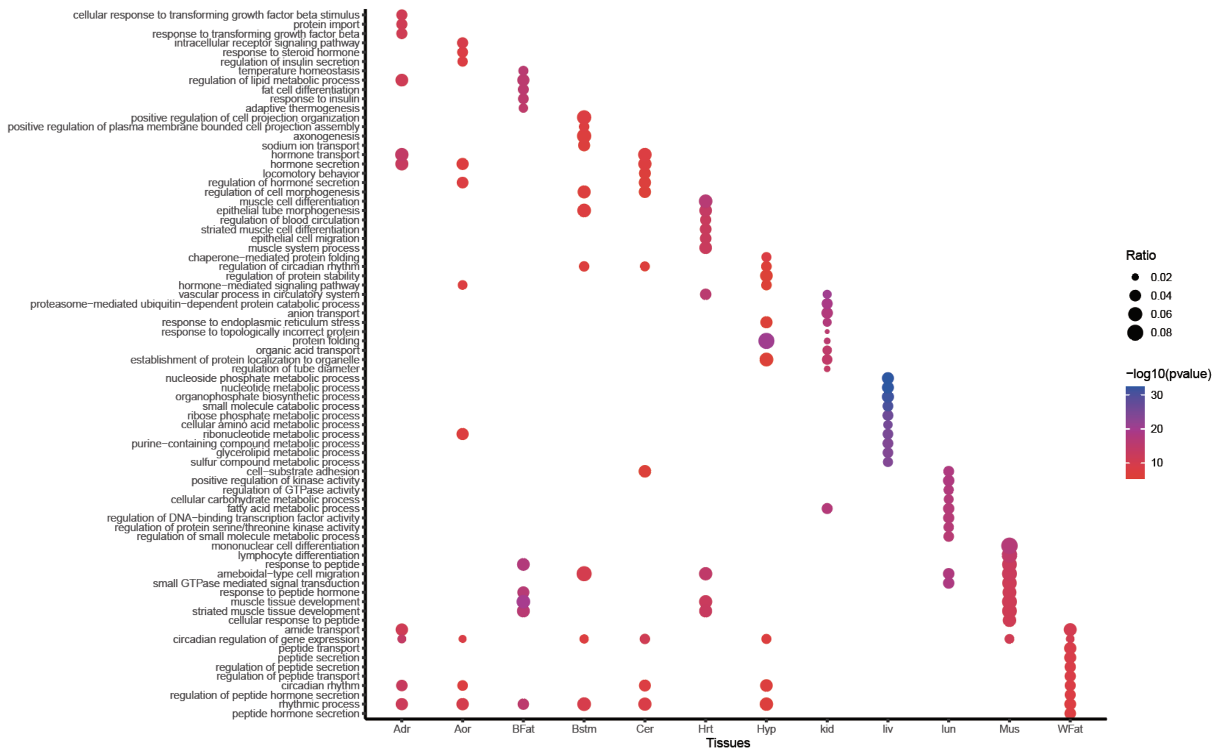

Figure S2. Functional annotation of circadian genes for mouse circadian atlas. Scatter plot displaying the top 10 Biological Processes for the mouse circadian atlas as determined by Gene Ontology (GO) annotation.

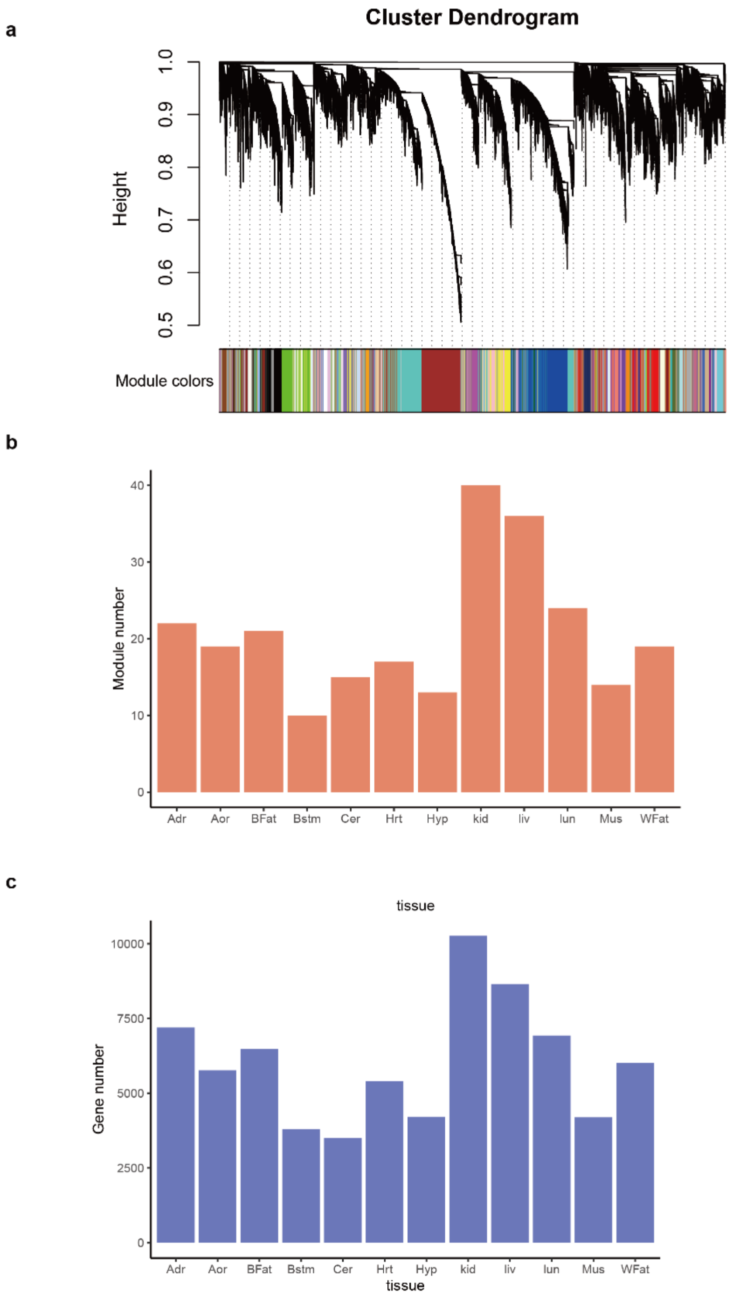

Figure S3. Weighted Gene Co-expression Network Analysis (WGCNA) of Mouse Circadian Atlas.

(a) Cluster diagram visualizing the co-expression network of the mouse circadian atlas.

(b) Bar chart indicating the number of modules enriched by circadian genes for the mouse circadian atlas.

(c) Bar chart displaying the gene count of modules enriched by circadian genes for the mouse circadian atlas.

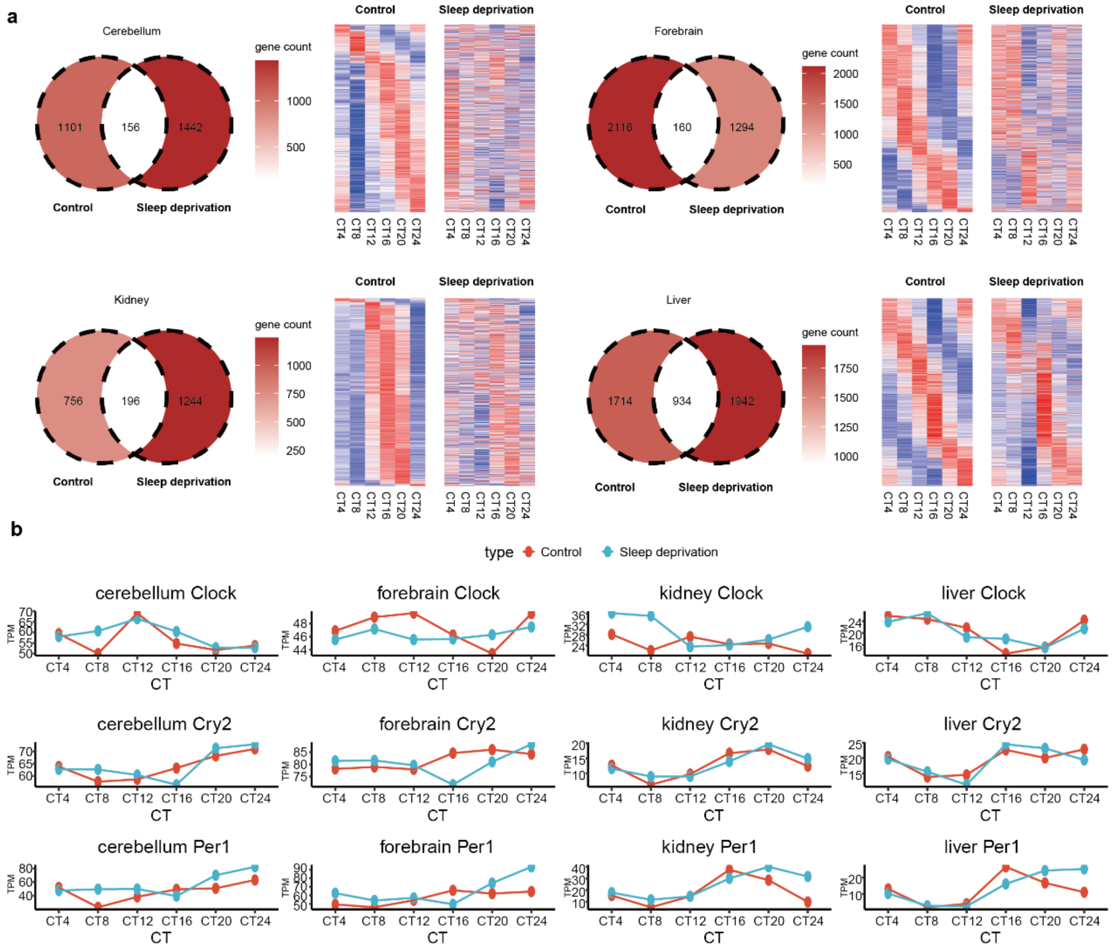

Figure S4. Circadian transcriptome analysis during sleep deprivation.

(a) Venn diagram illustrating the overlap of circadian genes affected by sleep deprivation. The heatmap displays the circadian expression pattern of control circadian genes under conditions of sleep deprivation.

(b) Scatter plots depicting the expression levels of core clock genes related to sleep regulation.

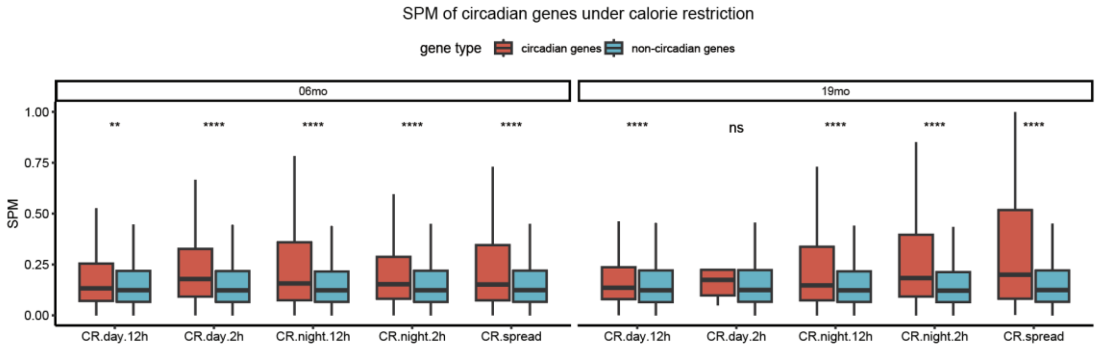

Figure S5. SPM of rhythmically expressed genes under calorie restriction.

Box plot depicting the tissue-specific values (SPM) of rhythmically expressed genes under conditions of calorie restriction.

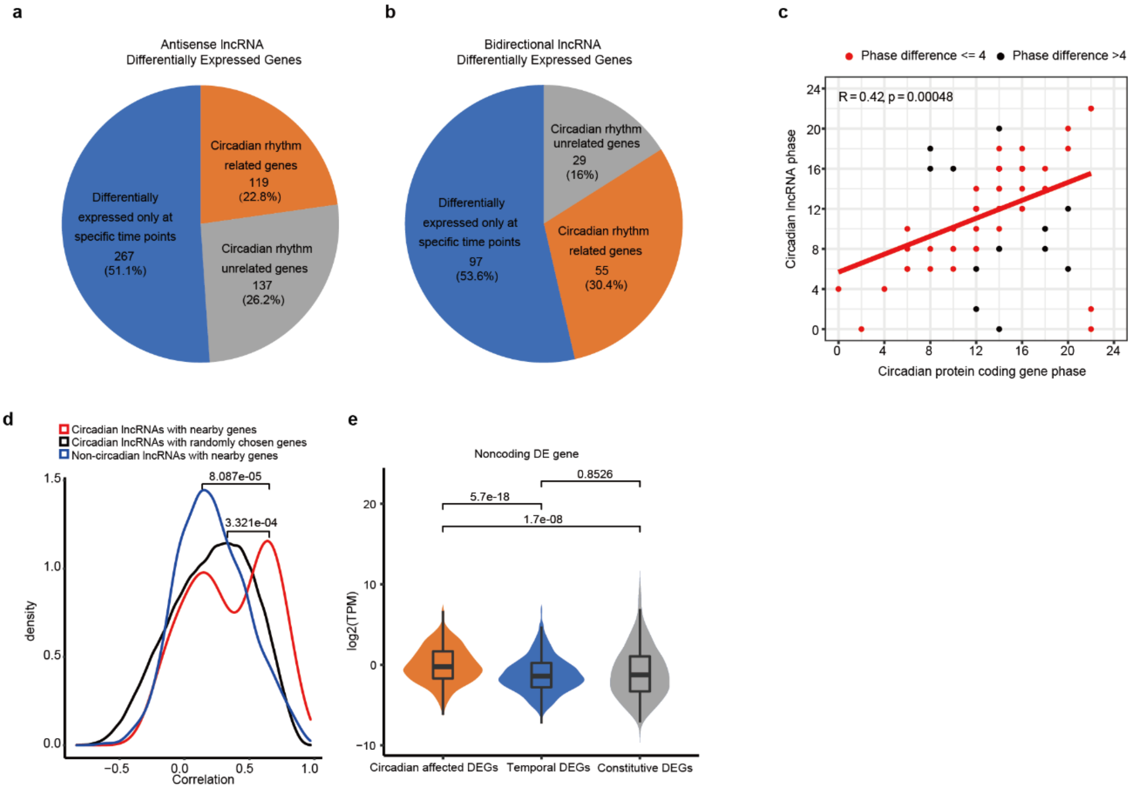

Figure S6. Temporal organization of differentially expressed lncRNAs (DE-lncRNAs) between liver and kidney

(a) The pie chart displays the proportion and number of three classes of differentially expressed antisense lncRNAs. Among all DE-lncRNAs, 22.8% are circadian-affected, 26.2% are constitutive, and 51.1% are temporal DEGs.

(b) The pie chart displays the proportion and number of three classes of differentially expressed bidirectional lncRNAs. Among all DE-lncRNAs, 30.4% are circadian-affected, 16% are constitutive, and 53.6% are temporal DEGs.

(c) Scatter plot of the phase correlation between circadian lncRNAs and nearby protein-coding genes.

(d) The density plot showed Pearson correlation coefficient between lncRNAs and nearby protein-coding genes. K-S test was used for distribution comparison

(e) Expression abundance comparison among the three classes of DE-lncRNAs. P values were determined by Wilcoxon rank sum test.

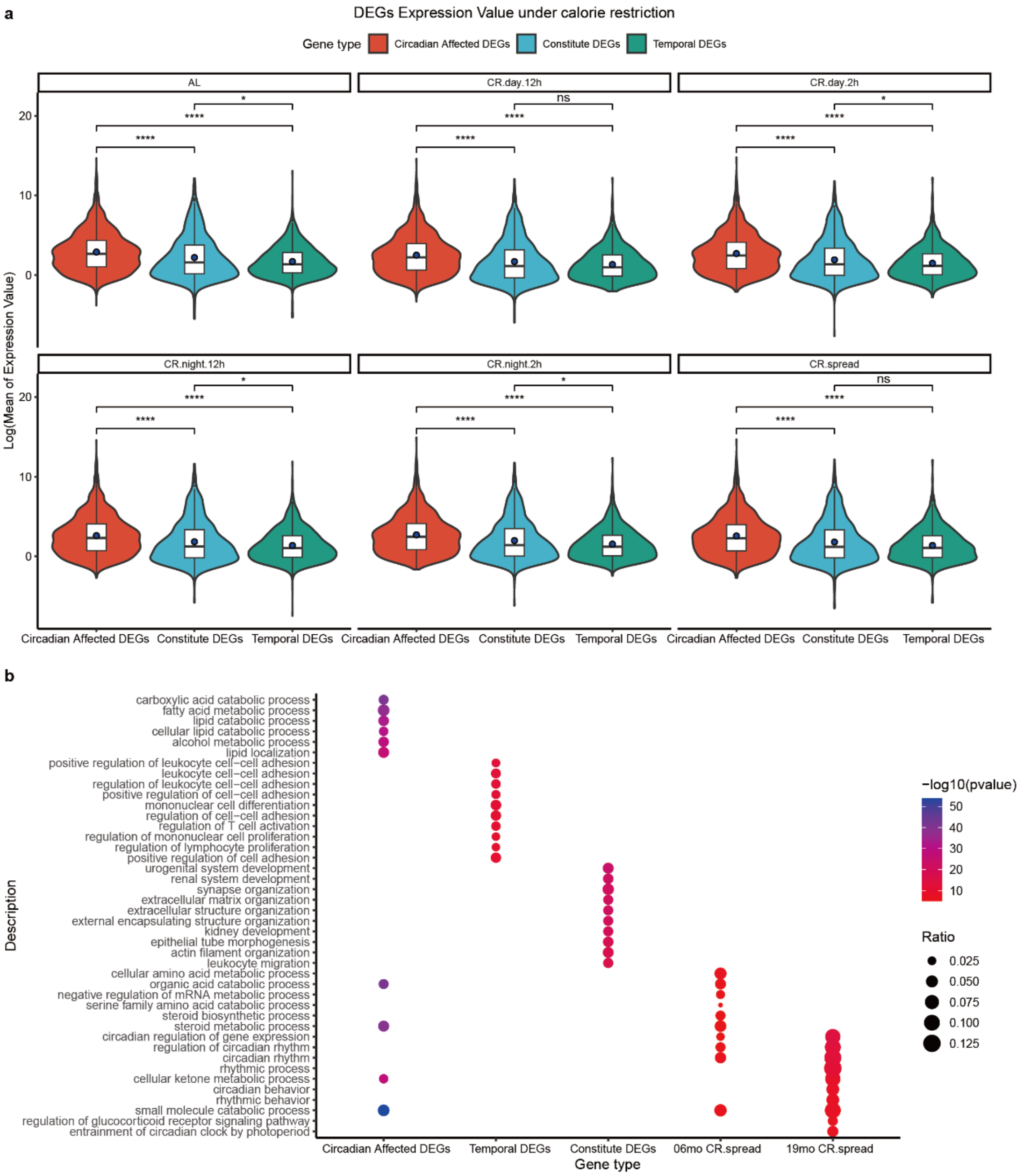

Figure S7. Analysis of three types of differentially expressed genes (DEGs) Under calorie restriction.

(a) Violin plot illustrating a comparison of gene expression levels among three types of DEGs under conditions of calorie restriction.

(b) Scatter plot displaying Gene Ontology (GO) functional annotations for three types of DEGs and circadian genes under caloric restriction (CR).

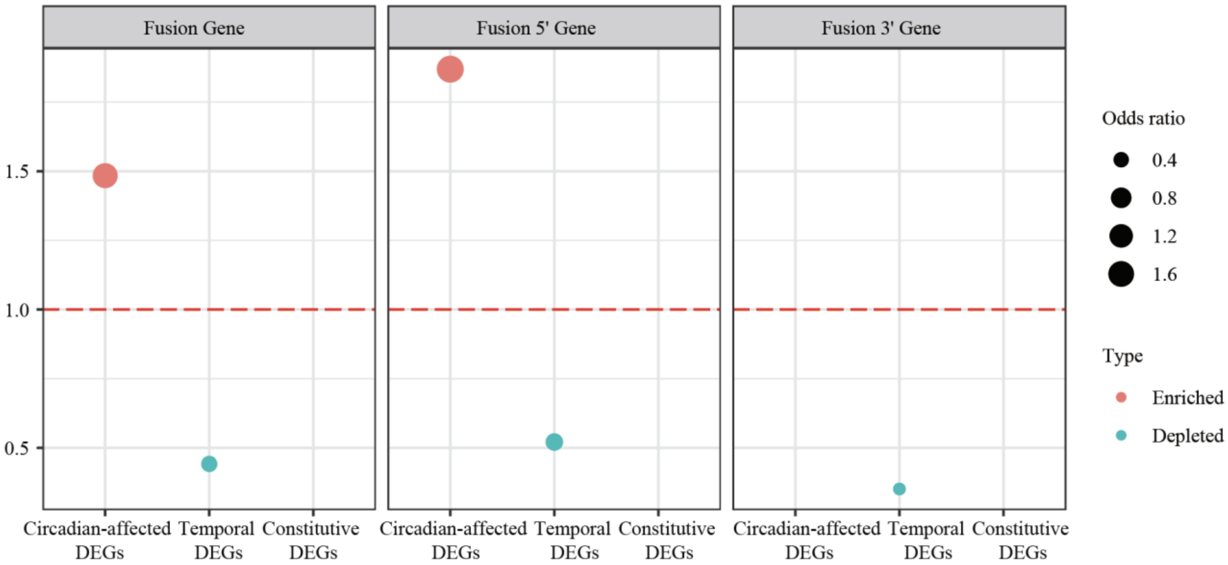

Figure S8. Enrichment analysis of three types of DEGs with fusion genes in cancer tissue.

Enrichment analysis of three types of DEGs with fusion genes from long-read sequencing data for the liver. The “enriched” and “depleted” relationships are shown in red and blue, respectively. The odds ratio is indicated by the size of the circle. Non-significance is indicated as blank.

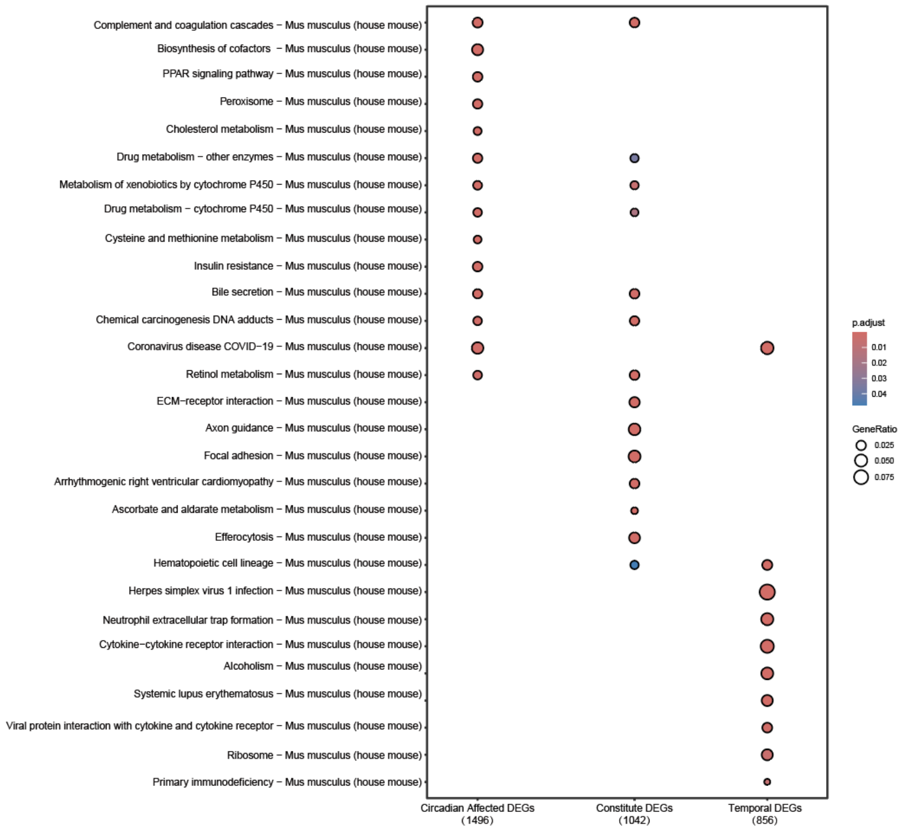

Figure S9. Functional annotation of the three classes of DEGs by KEGG analysis. Circadian-affected DEGs exhibit enrichment in metabolic functions, whereas other DEGs are associated with distinct biological pathways. Both enrichment odds ratio and significance level (P) are displayed.

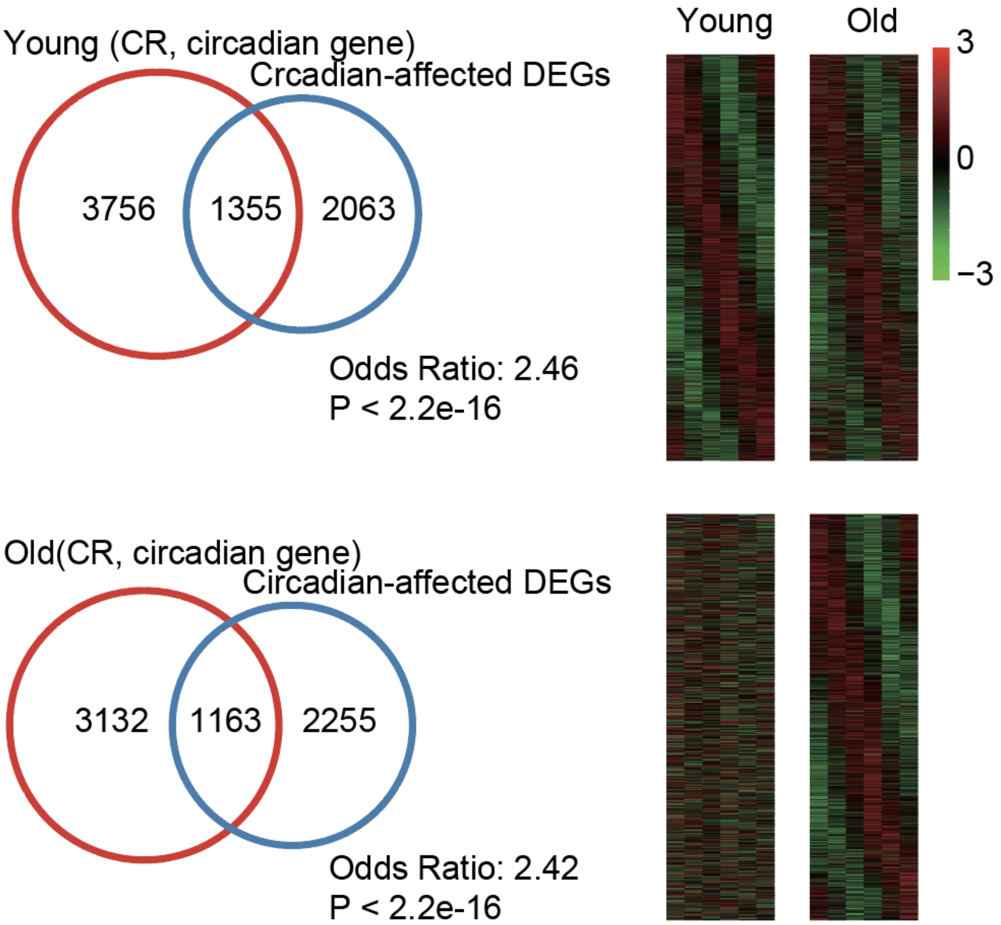

Figure S10. Enrichment analysis of circadian-affected DEGs and circadian genes under caloric restriction, both in young and old mice. Venn diagram showing the intersection of circadian-affected DEGs and circadian genes under caloric restriction(CR), both in young and old mice. Heatmap shows the circadian expression of overlapped genes.

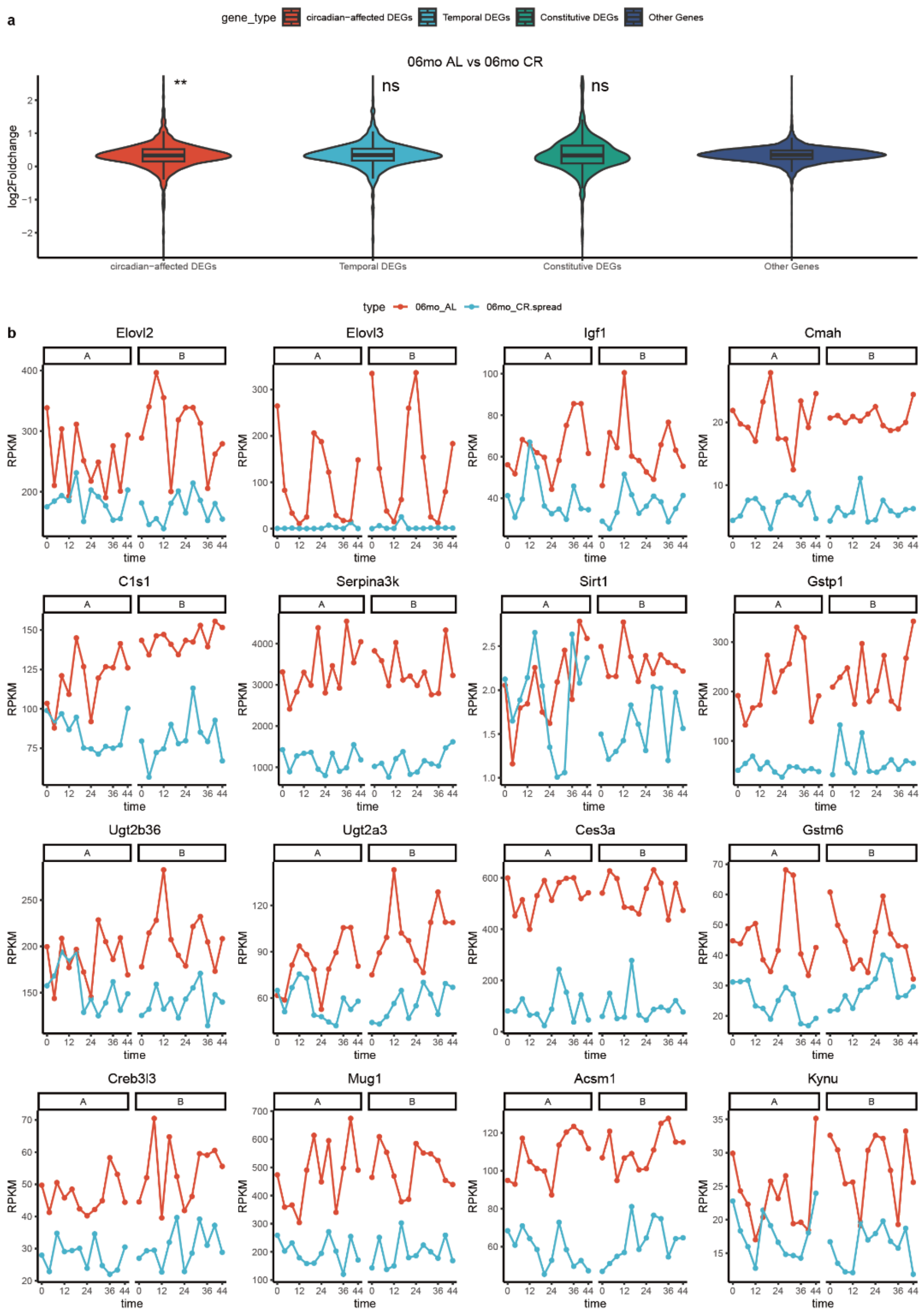

Figure S11. Relationship between circadian rhythm and aging genes under caloric restriction.

(a) Violin plot illustrating the comparison of gene log2 fold change among the three types of DEGs under conditions of young mice fed ad libitum and young mice under caloric restriction. P values were determined by the Wilcoxon rank sum test.

(b) Scatter plots displaying the comparison of Reads Per Kilobase Million (RPKM) values between aging genes and other genes under caloric restriction.

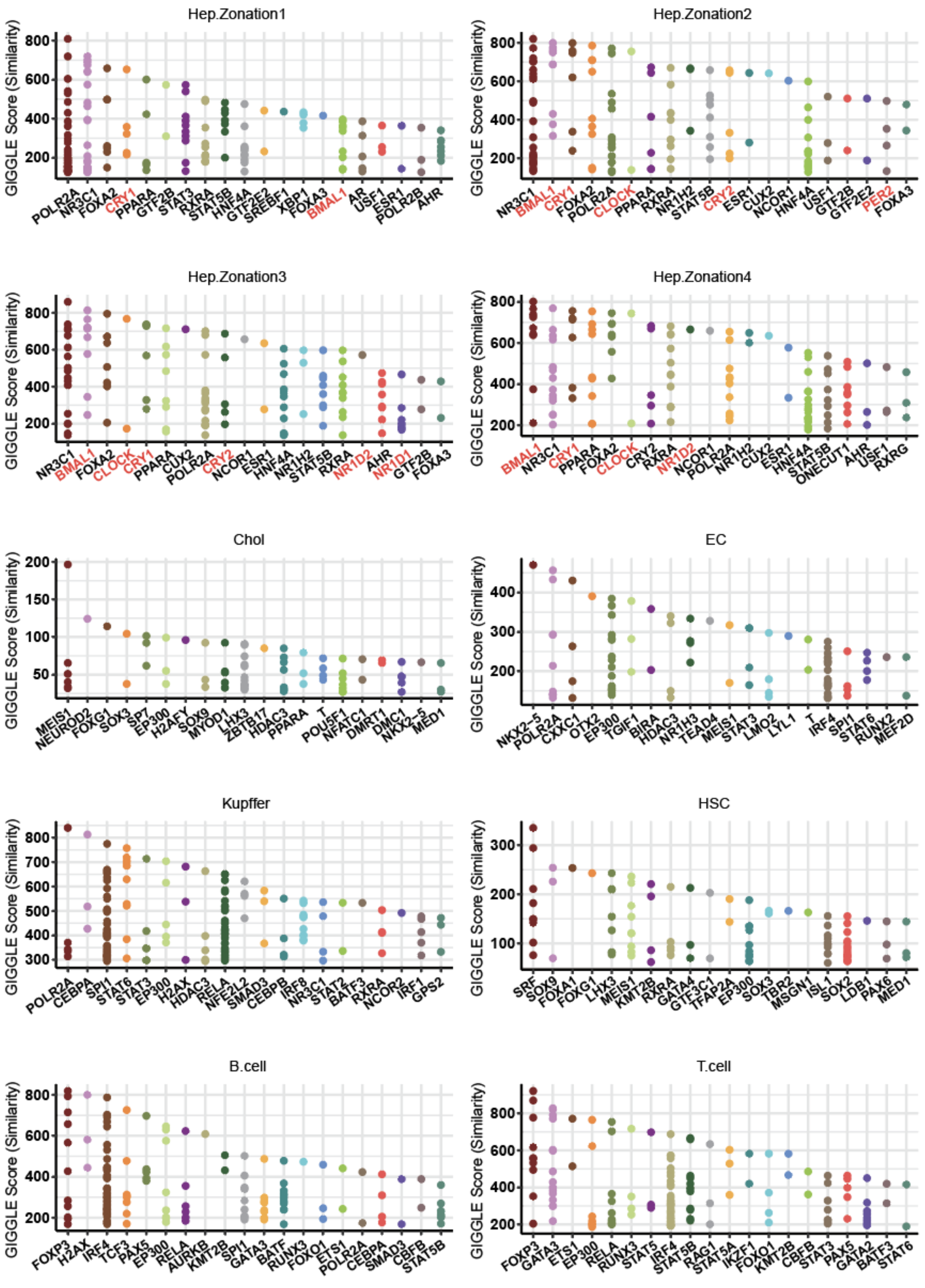

Figure S12. Potential transcription factors prediction of highly expressed genes for different cell types. Scatter plots showing Potential transcription factors by marker genes for different cell types**.**

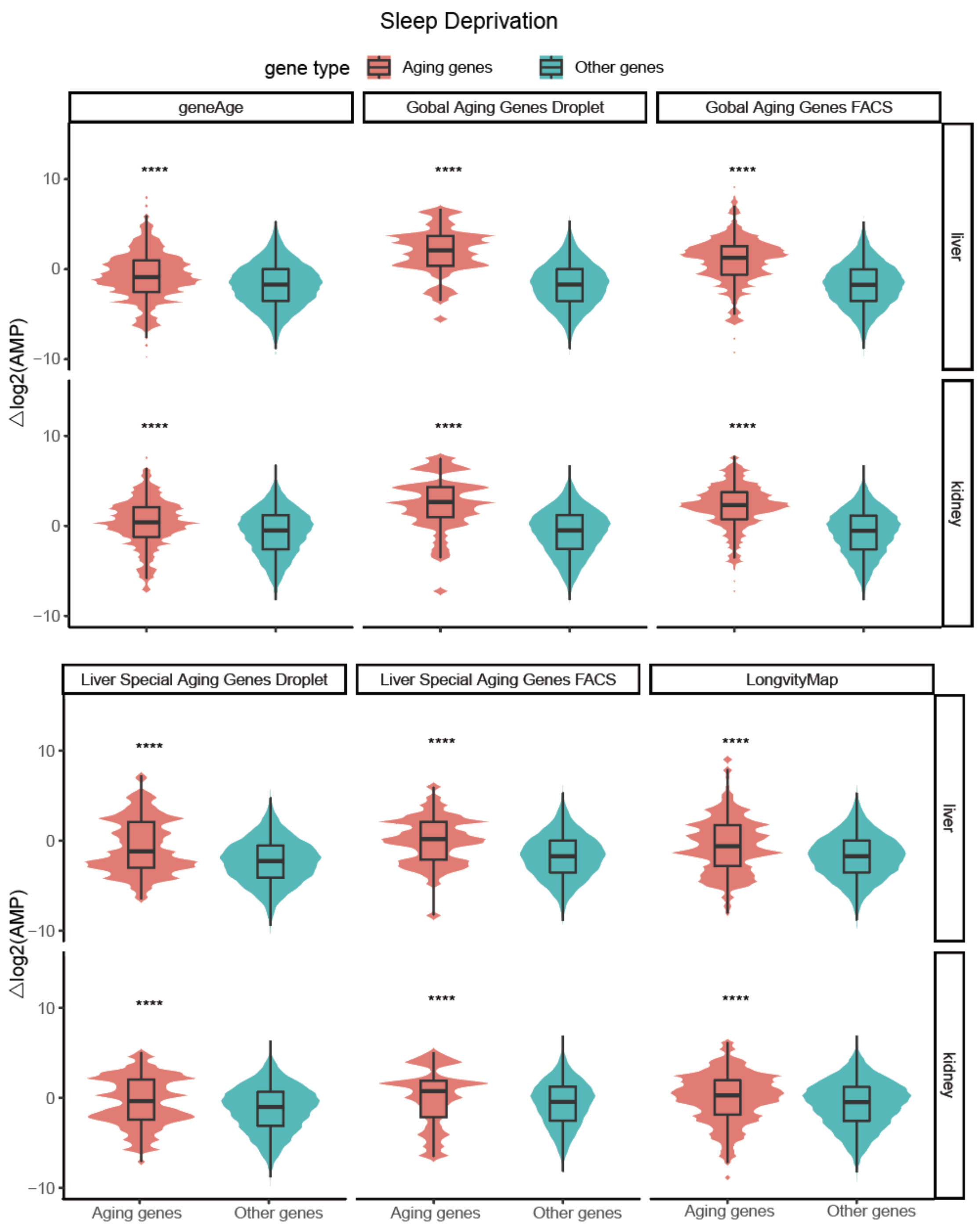

Figure S13. Disturbance of genes related to aging under sleep deprivation conditions. A violin plot illustrating the change in circadian amplitude (AMP) of aging and longevity genes in the liver and kidney, compared to other genes, during a 10-hour sleep deprivation period. All six aging and longevity gene lists are included in the plot.

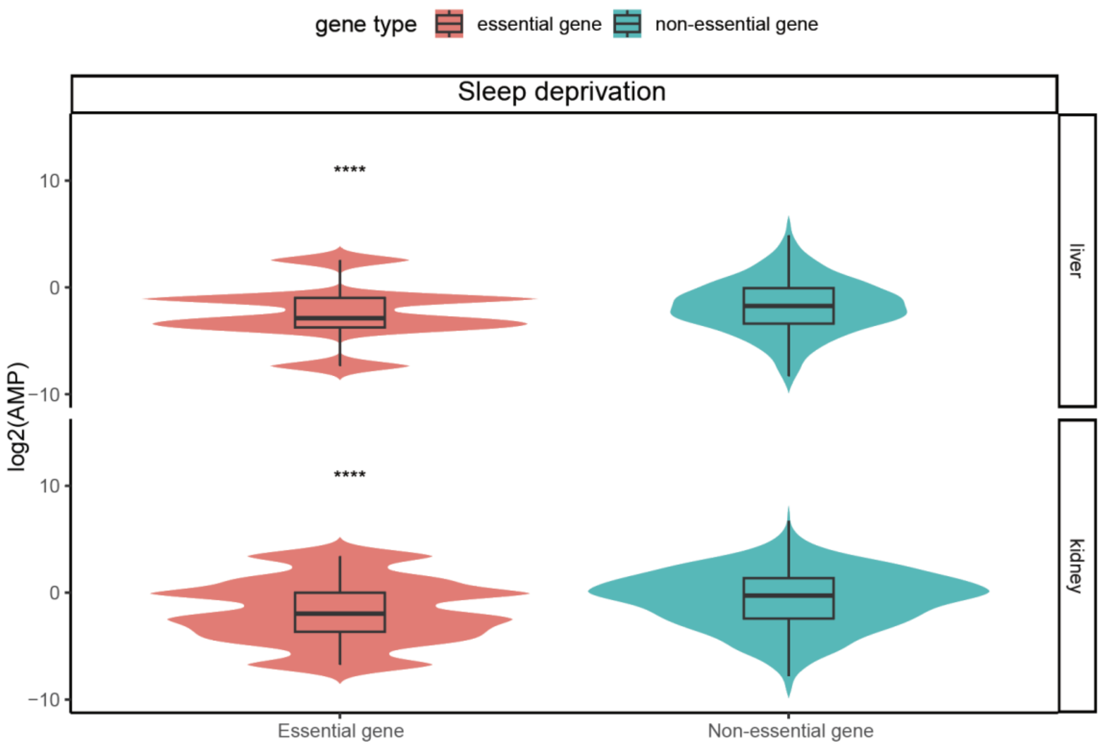

Figure S14. Stabilization of Essential Genes under Sleep Deprivation Conditions. A violin plot is utilized to demonstrate the change in circadian amplitude (AMP) of essential genes in the liver and kidney, compared to other genes, during a 10-hour sleep deprivation period.

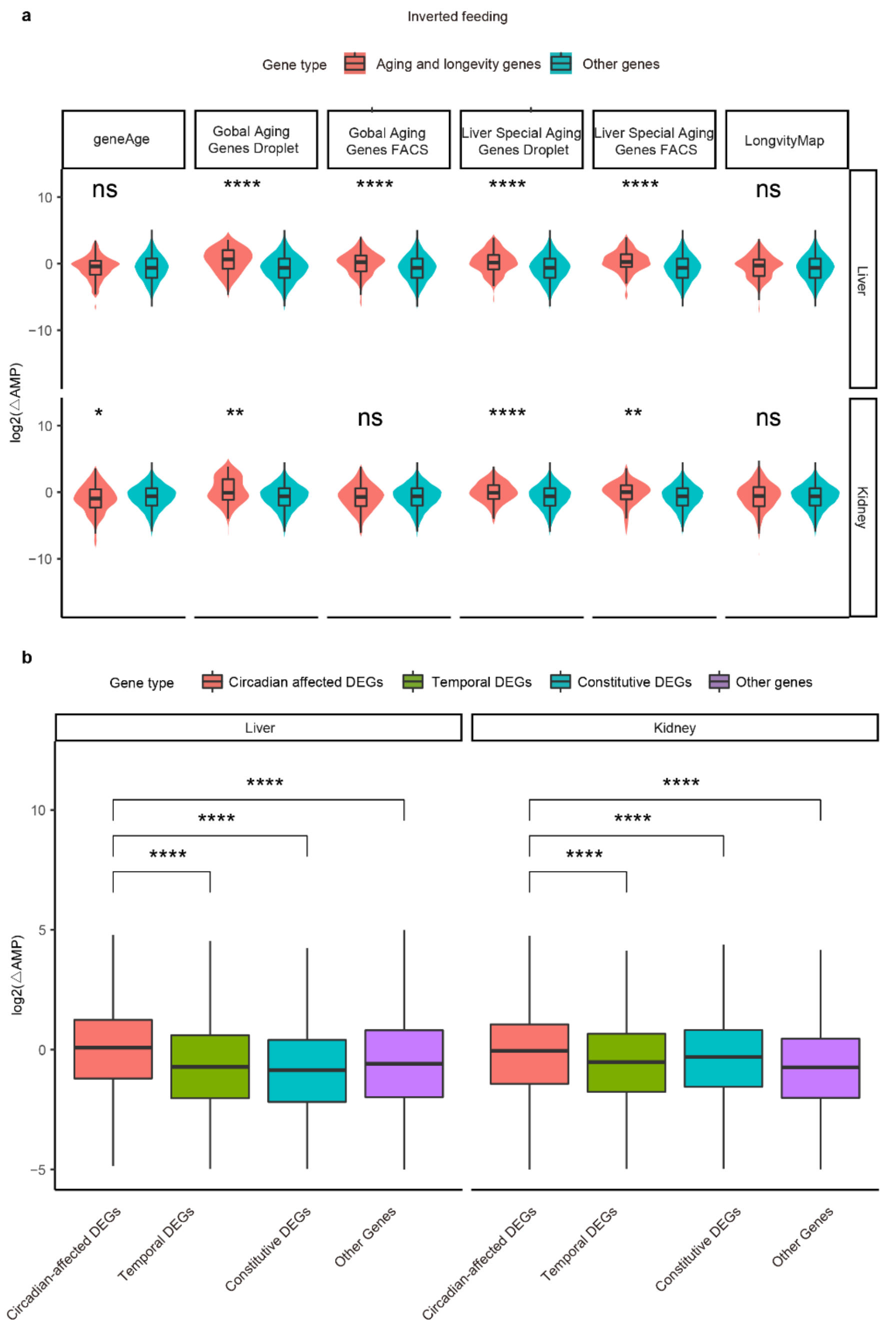

Figure S15. Genes related to aging are disturbed in inverted food intake condition

(a) A violin plot to show the change in circadian amplitude (AMP) of aging and longevity genes in the liver in comparison to other genes during inverted food intake condition. All 6 aging and longevity gene lists are included in the plot.

(b) A boxplot to compare the changes in circadian AMP for circadian-affected DEGs, constitutive DEGs, temporal DEGs, and other genes during inverted food intake condition. Both conditions exhibit similar trends. Statistical significance is denoted by ***, $P < 0.001$; **, $P < 0.01$; *, $P < 0.05$; and n.s. for non-significant differences.
