# Supplementary material for: Circadian-driven tissue specificity is constrained under caloric restricted feeding conditions
Source: Commun Biol. 2024 Jun 20;7:752. doi: 10.1038/s42003-024-06421-0 (PMC11190204; doi:10.1038/s42003-024-06421-0)
Supplement: Supplementary file 2 — Description of Additional Supplementary Files [file 42003_2024_6421_MOESM2_ESM.pdf]

Description of Additional Supplementary Files

File name: Supplementary Data 1
Description: List of Differentially Expressed Genes (DEGs) Identified in the Study.

File name: Supplementary Data 2
Description: Prediction of Transcription Factors for the Three Classes of DEGs.

File name: Supplementary Data 3
Description: Top 10 Biological Processes for DEGs by Gene Ontology (GO) Annotation.

File name: Supplementary Data 4
Description: Gene Sets Associated with Metabolism-Related Diseases and Cancer.

File name: Supplementary Data 5
Description: Gene Set Associated with Aging and Longevity.

File name: Supplementary Data 6
Description: Gene Set Associated with Caloric and Time Restriction-Related Circadian Regulation Only.

File name: Supplementary Data 7
Description: List of Genes in Liver Cycling Transcriptome Modules.
