# Supplementary material for: Circadian-driven tissue specificity is constrained under caloric restricted feeding conditions
Source: Commun Biol. 2024 Jun 20;7:752. doi: 10.1038/s42003-024-06421-0 (PMC11190204; doi:10.1038/s42003-024-06421-0)
Supplement: Supplementary file 10 — Reporting Summary [file 42003_2024_6421_MOESM10_ESM.pdf]

nature portfolio

Corresponding author(s): Guang-Zhong Wang

Last updated by author(s): May 13, 2024

Reporting Summary

Nature Portfolio wishes to improve the reproducibility of the work that we publish. This form provides structure for consistency and transparency in reporting. For further information on Nature Portfolio policies, see our Editorial Policies and the Editorial Policy Checklist.

Statistics

For all statistical analyses, confirm that the following items are present in the figure legend, table legend, main text, or Methods section.

n/a	Confirmed	
☐	☒	The exact sample size (*n*) for each experimental group/condition, given as a discrete number and unit of measurement
☐	☒	A statement on whether measurements were taken from distinct samples or whether the same sample was measured repeatedly
☐	☒	The statistical test(s) used AND whether they are one- or two-sided<br>*Only common tests should be described solely by name; describe more complex techniques in the Methods section.*
☐	☒	A description of all covariates tested
☐	☒	A description of any assumptions or corrections, such as tests of normality and adjustment for multiple comparisons
☐	☒	A full description of the statistical parameters including central tendency (e.g. means) or other basic estimates (e.g. regression coefficient) AND variation (e.g. standard deviation) or associated estimates of uncertainty (e.g. confidence intervals)
☐	☒	For null hypothesis testing, the test statistic (e.g. *F*, *t*, *r*) with confidence intervals, effect sizes, degrees of freedom and *P* value noted<br>*Give P values as exact values whenever suitable.*
☒	☐	For Bayesian analysis, information on the choice of priors and Markov chain Monte Carlo settings
☐	☒	For hierarchical and complex designs, identification of the appropriate level for tests and full reporting of outcomes
☐	☒	Estimates of effect sizes (e.g. Cohen's *d*, Pearson's *r*), indicating how they were calculated

Our web collection on statistics for biologists contains articles on many of the points above.

Software and code

Policy information about availability of computer code

Data collection	Gene expression data for its own experiment. All raw and processed sequencing data generated in this study have been submitted to the NCBI Gene Expression Omnibus (GEO; https://www.ncbi.nlm.nih.gov/geo/) under accession number GSE240693.
Data analysis	We used the Jonckheere-Terpstra-Kendall algorithm from the MetaCycle R package (version 1.20) to identify the oscillations of genes, differential expression analysis was performed using both DESeq2 (version 1.26.0) and EdgeR (version 3.28.1). We calculated Tau for each gene to evaluate tissue-specificity of the transcriptome . And we also used an additional tissue-specificity indicator, expression enrichment (EE), to validate our main discoveries. We utilized WCGNA(version1.69) to build weighted co-expresion network in R (version 4.0.2)

For manuscripts utilizing custom algorithms or software that are central to the research but not yet described in published literature, software must be made available to editors and reviewers. We strongly encourage code deposition in a community repository (e.g. GitHub). See the Nature Portfolio guidelines for submitting code & software for further information.

Data

Policy information about availability of data

All manuscripts must include a data availability statement. This statement should provide the following information, where applicable:

- Accession codes, unique identifiers, or web links for publicly available datasets
- A description of any restrictions on data availability
- For clinical datasets or third party data, please ensure that the statement adheres to our policy

All raw and processed sequencing data generated in this study have been submitted to the NCBI Gene Expression Omnibus (GEO; https://www.ncbi.nlm.nih.gov/geo/) under accession number GSE240693.
To identify circadian genes under caloric restriction conditions, we using data from GSE57830 and GSE190939.
Liver single cell data from GSE143526.
Metabolic-related diseases were identified based on a previous study (Zhou et al. 2020).Disease genes associated with metabolism were extracted from the Human Gene Mutation Database (HGMD) and Cancer Genome Atlas (TCGA, https://portal.gdc.cancer.gov/).
Aging-related diseases from a previous report (Zhang et al. 2021). Related disease gene sets were obtained from the MalaCards Human Disease Database.
Aging and longevity genes contain three independent gene collections from the Tabula Muris Senis single-cell dataset (Schaum et al. 2020) , LongvityMap database and GeneAge databases.

Research involving human participants, their data, or biological material

Policy information about studies with human participants or human data. See also policy information about sex, gender (identity/presentation), and sexual orientation and race, ethnicity and racism.

Reporting on sex and gender	NA
Reporting on race, ethnicity, or other socially relevant groupings	NA
Population characteristics	NA
Recruitment	NA
Ethics oversight	NA

Note that full information on the approval of the study protocol must also be provided in the manuscript.

Field-specific reporting

Please select the one below that is the best fit for your research. If you are not sure, read the appropriate sections before making your selection.

☒ Life sciences ☐ Behavioural & social sciences ☐ Ecological, evolutionary & environmental sciences

For a reference copy of the document with all sections, see nature.com/documents/nr-reporting-summary-flat.pdf

Life sciences study design

All studies must disclose on these points even when the disclosure is negative.

Sample size	For differential expression of liver and kidney, we collected four replicates of liver and kidney every four hours throughout the day and we collected liver and kidney samples from the same mice at each sampling time, 48 samples in total. About the sleep deprivation experiment, we collected two replicates of 4 peripheral circadian tissues (forebrain, cerebellum, liver, kidney) were obtained at next six circadian time (CT) points with an interval of 4 h (CT4, CT8, CT12, CT16, CT20, and CT24), 98 samples in total.
Data exclusions	No data were excluded from the analyses.
Replication	For differential expression of liver and kidney, each organization has four biological repetition at each point of time. For sleep deprivation experiments, each organization contains two biological repetition at each point of time.
Randomization	The mouse samples used were randomly assigned to the experimental group.
Blinding	Blinding was not relevant for the study. All group assignments were known based on known tissue.

Reporting for specific materials, systems and methods

We require information from authors about some types of materials, experimental systems and methods used in many studies. Here, indicate whether each material, system or method listed is relevant to your study. If you are not sure if a list item applies to your research, read the appropriate section before selecting a response.

Materials & experimental systems

n/a	Involved in the study
☒	☐ Antibodies
☒	☐ Eukaryotic cell lines
☒	☐ Palaeontology and archaeology
☐	☒ Animals and other organisms
☒	☐ Clinical data
☒	☐ Dual use research of concern
☒	☐ Plants

Methods

n/a	Involved in the study
☒	☐ ChIP-seq
☒	☐ Flow cytometry
☒	☐ MRI-based neuroimaging

Animals and other research organisms

Policy information about studies involving animals; ARRIVE guidelines recommended for reporting animal research, and Sex and Gender in Research

Laboratory animals	The C57/BL6 male mice used in this study were raised in a laboratory with a 12-hour light-dark cycle (lights on at 8 a.m. and off at 8 p.m.). After entrainment to this cycle for five days, the mice were transferred to constant darkness (DD) and sacrificed 24 hours later. The sleep deprivation experiment followed previously reported protocols (Lu et al. 2021). Mice were randomly assigned to either the experimental or control group. The experimental group was subjected to a sleep deprivation instrument, which involved forced locomotion through a slowly rotating drum (40 cm in diameter, 0.4 m per min) while having access to food and water. All mice in experimental group were placed in the sleep deprivation device for 10 h (6:00 AM–4:00 PM) during their normal resting phase while the control animals were undisturbed.
Wild animals	The study did not involve wild animals.
Reporting on sex	We used C57BL/6J male mice; however, there could be strain and sex-specific responses worth studying further
Field-collected samples	This study does not involve field work.
Ethics oversight	All animal experiments were approved by the Animal Care and Use Committee of the Shanghai Institute of Nutrition and Health and were conducted in accordance with institutional guidelines.

Note that full information on the approval of the study protocol must also be provided in the manuscript.
